# Effect of Acidic Industrial Effluent Release on Microbial Diversity and Trace Metal Dynamics During Resuspension of Coastal Sediment

**DOI:** 10.3389/fmicb.2018.03103

**Published:** 2018-12-14

**Authors:** Hana Zouch, Léa Cabrol, Sandrine Chifflet, Marc Tedetti, Fatma Karray, Hatem Zaghden, Sami Sayadi, Marianne Quéméneur

**Affiliations:** ^1^Laboratory of Environmental Bioprocesses, Biotechnology Center of Sfax, Sfax, Tunisia; ^2^Aix Marseille Univ, Université de Toulon, CNRS, IRD, MIO UM 110, Marseille, France

**Keywords:** trace metals, marine sediments, acidic wastewater, phosphogypsum, bacteria, *Flavobacteriaceae*, Gulf of Gabès, Mediterranean Sea

## Abstract

Both industrial effluent discharge and the resuspension of contaminated marine sediments are important sources of trace metals in seawater which potentially affect marine ecosystems. The aim of this study was to evaluate the impact of the industrial wastewaters having acidic pH (2–3) and containing trace metals on microbial diversity in the coastal ecosystem of the Gulf of Gabès (Tunisia, southern Mediterranean Sea) subjected to resuspension events of marine sediments. Four trace elements (As, Cd, U, and V) were monitored during 10-day sediment resuspension experiments. The highest enrichment in the seawater dissolved phase was observed for Cd followed by U, V, and As. Cd remobilization was improved by indigenous microbial community, while U release was mainly abiotic. Acidic effluent addition impacted both trace metal distribution and microbial diversity, particularly that of the abundant phylum *Bacteroidetes*. Members of the order *Saprospirales* were enriched from sediment in natural seawater (initial pH > 8), while the family *Flavobacteriaceae* was favored by acidified seawater (initial pH < 8). Some *Flavobacteriaceae* members were identified as dominant species in both initial sediment and experiments with acidic wastewater, in which their relative abundance increased with increasing dissolved Cd levels. It could be therefore possible to consider them as bioindicators of metal pollution and/or acidification in marine ecosystems.

## Introduction

For several decades, many industrial complexes have settled on the coast of the Gulf of Gabès (GG), a shallow gulf located in the southern Mediterranean Sea. These coastal industrial expansions, coupled with urban growth, have enhanced marine pollution, mainly due to the discharge of urban/industrial effluents into seawater, which has led to a high diversity of contaminants, including metals (Ayadi et al., 2015; Zaghden et al., 2016; El Zrelli et al., 2018; Naifar et al., 2018). The long-term discharge of phosphogypsum (i.e., a by-product of the phosphate fertilizer industries having acidic pH and containing trace metals) and/or untreated acidic wastewaters in the GG has resulted in a progressive degradation and loss of biodiversity, which represent a real threat for the marine ecosystems (El Zrelli et al., 2017; El Kateb et al., 2018; Naifar et al., 2018). Recent studies have reported links between high metal levels in coastal sediments and different marine organisms of GG (Gargouri et al., 2011; Ghannem et al., 2014; El Zrelli et al., 2015; Rabaoui et al., 2015). Due to their toxicity, long-term persistence and undegradability, metals in marine ecosystems may also pose a potential human health risk through their transfer, accumulation in the food chain and subsequent consumption.

Generally, sediments act as an important sink for trace metals, thus reducing their bioavailability in marine ecosystems (Eggleton and Thomas, 2004; Tessier et al., 2011). In sediment, trace metals can be adsorbed to amorphous materials, complexed with organic matter, or present in secondary minerals (Peng et al., 2009). However, their transfer into seawater is regulated by hydrodynamics, biogeochemical and physicochemical factors (Eggleton and Thomas, 2004; Tessier et al., 2011). Thus, sediments can become a potential source of metals for seawater via remobilization processes (Saulnier and Mucci, 2000; Kim et al., 2006; Kalnejais et al., 2010; Xu et al., 2015). Marine sediment resuspension may occur: (i) during natural events, such as tides, waves, storms, and biological activities, or (ii) through human activities, such as dredging, vessel movements and fishing (Eggleton and Thomas, 2004; Gadd, 2010). The GG displays the highest tides in the Mediterranean Sea (up to 2.3 m, Sammari et al., 2006), due to its large continental shelf with a very low slope. Surface sediment resuspensions induced by tides and currents is one of the main assumptions to explain the continuous nutrient supply in GG shallow waters (Hassen et al., 2009; Rekik et al., 2012; Hamdi et al., 2015), which are considered as one of the most productive areas of the Mediterranean Sea (Mayot et al., 2016; Ayata et al., 2018). Besides nutrients, recent studies have suggested that the surface sediment resuspension in the GG may be a significant source of metals (Ben Salem and Ayadi, 2016), which may also influence biological activities in these shallow waters (in addition to industrial activities).

Microorganisms play a fundamental role in marine ecosystem functioning. Changes in environmental conditions (e.g., pH, nutrients), as well as chemical contaminants (e.g., metals) entering the marine environment, can modify their diversity and ecological functions (Gillan et al., 2005; Witt et al., 2011; Wang K. et al., 2015; Goni-Urriza et al., 2018). Microbial communities can be used as indicators of contaminant stress because they are highly sensitive to slight changes in their surrounding environment (Sun et al., 2012). Microorganisms can also play an important role in the metal mobility through different processes (e.g., oxidation/reduction reactions or organic/inorganic acid formation), therefore increasing their bioavailability (Gadd, 2004). For instance, both iron- and sulfur-oxidizing bacteria can release soluble metals from solid metal-bearing phases (Tabak et al., 2005; Fonti et al., 2013). On the contrary, many microorganisms can contribute to metal immobilization by biosorption, transport, and intracellular sequestration, or precipitation, thus reducing their bioavailability (White et al., 1997; Gadd, 2004). For instance, sulfate-reducing bacteria are strongly involved in metal immobilization by the precipitation of metal sulfide in sediments (Gadd, 2000; Jong and Parry, 2003). However, the microbial populations associated with metal remobilization during surface sediment resuspension events have not been well identified in coastal and marine ecosystems, whereas metal remobilization has been extensively investigated from estuarine or marine sediments (Cantwell and Burgess, 2004; Shipley et al., 2011; Dang et al., 2015). Moreover, the impact of acidic industrial effluent discharge (especially industrial waste containing metals) on the microbial diversity of coastal marine ecosystems has never been studied to date.

In this work, we evaluated for the first time the effect of acidic and metal-rich wastewaters (AWW) as released by fertilizer industries on both microbial diversity and trace elements (TE; i.e., trace metals, metalloids, and radionuclides) during experimental resuspension of contaminated surface coastal sediments. The bacterial community dynamics and remobilization of four TE (arsenic, As; cadmium, Cd; uranium, U; vanadium, V) were specifically monitored over time. The potential impact of microbial communities on TE mobility was also examined by comparing biotic experiments and abiotic controls to distinguish between biotic and abiotic processes involved in TE mobility. Both the metalloid As and the metal Cd could come from all of the various anthropogenic sources referenced by Ross (1994). Phosphate fertilizer industries are also known as important sources of the toxic metal Cd and the radionuclide U (Yamazaki and Geraldo, 2003; Cichy et al., 2014). In turn, V represents one of the most abundant metals in petroleum and can be used as a tracer of oil pollution in coastal environments (Guzmán and Jarvis, 1996), such as the hydrocarbon-impacted coastal areas of GG (Fourati et al., 2018a,b).

## Materials and Methods

### Studied Area

**Figure 1:**
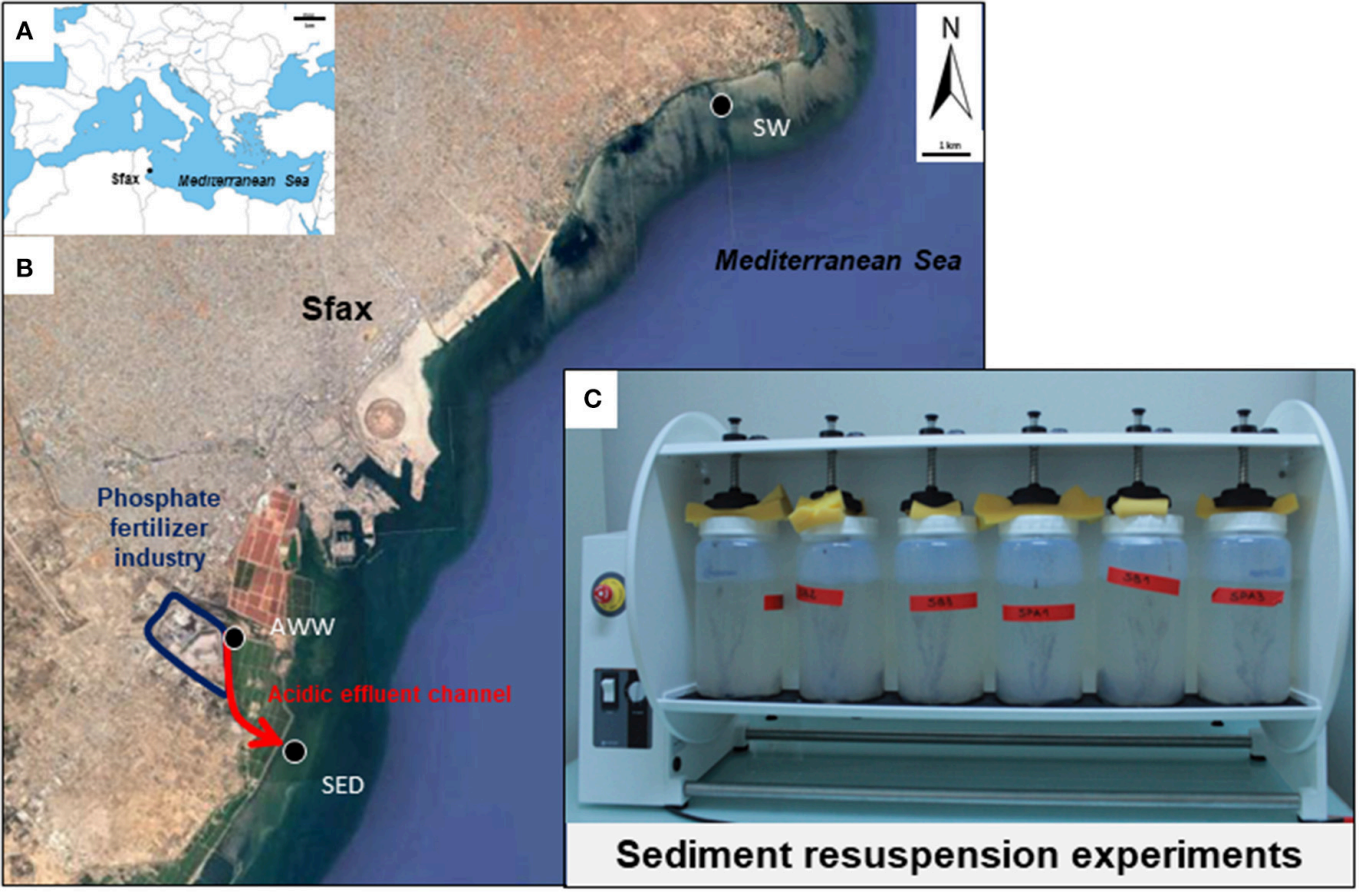
Map of the study area in the south Mediterranean Sea **(A)** and in the Sfax coast **(B)** and photograph of the laboratory experiments of sediment resuspension **(C)**. Location of sampling sites is shown by black circles. Location of the fertilizer plant in the industrial area is indicated in blue. Red arrows indicate the direction of flow of acidic wastewater.

Sfax (34°43′N−10°46′E) is the second largest city in Tunisia and is located in the northern part of the Gulf of Gabès (GG, 100 km wide from Sfax to Djerba and 100 km long from Gabès to the open Mediterranean Sea, Figure 1A). The southern coast of Sfax city is impacted by numerous polluting industrial plants, such as the phosphate fertilizer industry (Figure 1B).

Phosphogypsum produced by the Industrial Society of Phosphoric Acid and Fertilizer (SIAPE) was stored in stockpiles (tabia) in front of Sfax coast since the 80th's, which resulted in the drainage of a liquid having acidic pH (abbreviated in this paper as acidic wastewater, AWW; flow rate of ~500 m^3^ d^−1^). This AWW was mixed with the treated domestic wastewaters of the National Sanitation Agency (ONAS; flow rate of ~45000 m^3^ d^−1^), olive oil wastewaters of storage basins and the Thyna municipal landfill leaching waters through a channel “El Hakmouni” before sea outfall. Production of phosphoric acid by the SIAPE stopped since august 2016 and no phosphogypsum was produced since this date (Chemical Group of Tunisia, personal communication).

### Sample Collection, Processing, and Storage

Acidic wastewater (AWW), contaminated marine sediment (SED) and seawater (SW) were sampled on July 4th 2016 on the coast of Sfax (GG, Tunisia, southern Mediterranean Sea; see location of sampling sites on Figure 1).

All bottles were pre-cleaned following a rigorous protocol: filled with 10% HCl (VWR Analytical grade, 1 week) rinsed with ultra-pure MilliQ water (R = 18.2 MΩ.cm^−1^) filled with 2% HCl (Fisher, Optima grade) and stored in closed plastic bags until use.

For laboratory experiments, AWW was collected using sterile plastic bottles at a point (34°42′05.23′′N−10°44′02.65′′E) above its mixture with different wastewaters and discharge into the seawater, to distinguish the specific effect of phosphogypsum leachates from mixed wastewaters. Surface SED affected by resuspension events (0–5-cm layer) was sampled at low tide, at 10 m from the shore, near the discharge of mixed wastewaters into the seawater (34°40′46.95′′N−10°44′45.74′′E) with a plastic spatula and distributed in sterile plastic bags (~1 kg). In order to assess the environmental impact due to AWW discharge during SED resuspension experiments by coastal seawater (described below), SW was taken at high tide (0.5 m below sea level) on the northern coast of Sfax at a point less impacted by the southern fertilizer industry (34°47′41.74′′N−10°51′05.00′′E) using 4-L pre-cleaned Polycarbonate bottles (Nalgene®). The temperature, pH, and redox potential of AWW, SED and SW were measured *in situ* using the multiparameter probe PC-5 (XS-Instruments; Table S1).

For TE analysis, AWW and SW samples were transferred in triplicate in commercial metal-free polypropylene tubes (VWR) previously washed with HCl 10%, and immediately filtered on 0.2-μm sterile cellulose acetate filters using pre-cleaned Minisart syringes. Back in the laboratory, they were transferred to pre-cleaned FEP bottles, acidified with 2% HNO_3_ (Fisher Optima grade) and stored separately in closed plastic bags at 4°C until total TE dissolved analysis.

For DNA extraction, SED subsamples were transferred in duplicate sterile Eppendorf tubes and 2 L of water samples (SW and AWW) were filtered in duplicate using 0.22-μm sterile cellulose ester filters (Millipore). Then, SED and filters (SW and AWW) were stored at −20°C, prior to molecular analysis.

### Sediment Analysis and granulometry

SED samples were freeze-dried and sieved onto 2 mm (10 g for granulometry) or 200 μm (1 g for total dissolved TE and elemental composition analyses). The water content of SED samples was determined after drying for 24 h at 105°C. Total carbon, hydrogen, nitrogen and sulfur (C/H/N/S) and organic C contents of dry SED were determined as previously described by Zouch et al. (2017). The granulometry of dry SED was determined with a Beckman Coulter LS 13 320 laser granulometer before and after organic matter removal and the relative abundance of sand (2000 to 63 μm), silt (63 to 2 μm), and clay (< 2 μm) was measured (Ghilardi et al., 2012). Before laser granulometry analysis, sediments must be sieved on 2 mm, which is the upper limit of the granulometer (Ghilardi et al., 2012; Guigue et al., 2017).

### Sediment Resuspension Experiments

Table 1 shows the four treatments tested in triplicate in the sediment resuspension experiments. In all conditions tested, SED was mixed with SW using a solid/liquid ratio of 10 g L^−1^ of dry sediment, a ratio close to *in situ* levels of suspended particulate matter measured during sediment resuspension events induced by many operations, such as dredging (Shipley et al., 2011; Monnin et al., 2018). To obtain this ratio, wet SED samples (~32.6 g corresponding to 20 g dry weight) were transferred into pre-cleaned mesocosms (2.2 L FEP bottles, Nalgene), and filled with SW up to 2 L.

**Table 1:** Treatments used to test the effect of acidic wastewater (AWW) on microbial and trace metal dynamics during the resuspension of contaminated sediment (SED) into coastal seawater (SW).

**Conditions**	**Name**	**Replicates**	**Sediment (SED)**	**Seawater (SW)**	**Acidic wastewater (AWW)**	**Poison (NaN_**3**_)**
Biotic conditions	S	S1, S2, S3	20 gDW	2L	0	0
	P	P1, P2, P3	20 gDW	2L	10 mL	0
Abiotic controls	SA	SA1, SA2, SA3	20 gDW	2L	0	50 mM
	PA	PA1, PA2, PA3	20 gDW	2L	10 mL	50 mM

*Experiments were performed in triplicates*.

To evaluate the effect of acidic effluent discharge on TE and microbial dynamics during sediment resuspension by seawater, two biotic conditions were compared: S condition without AWW addition and P condition with addition of 10 mL of AWW into 2-L mixture. Each condition was prepared in triplicate (Table 1). Resulting initial pH (7.0 for P and 8.5 for S) are close to *in situ* seawater pH values measured near the effluent discharge (pH 7 at SED point) and from coastal seawater unaffected by AWW (pH 8.3 at SW point). P conditions simulated the release of acidic wastewater effluent into the Sfax coastal ecosystem (with AWW input), while S conditions mimic coastal ecosystem after stopping effluent discharge (without AWW input).

Biotic and abiotic experiments were compared in order to distinguish between biotic and abiotic processes involved in TE mobility, and therefore to identify the potential impact of microbial communities on TE mobility. Abiotic controls (SA and PA) were prepared in triplicate by poisoning the sediment suspensions with sodium azide (NaN_3_) at a final concentration of 50 mM, which inhibits microbial growth and activity, as previously defined by Cabrol et al. (2017).

All mesocosms (*n* = 12 in total) were run in parallel and incubated aerobically undergoing continuous overhead shaking (10 tr.min^−1^, Heidolph Reax 20) to mimic sediment resuspension, for 10 days at 27°C (Figure 1C, Table 1).

Sediment/water mix samples (40 mL) were taken from the 12 mesocosms at 9 different times over the course of the resuspension experiment: 0, 1 h, 5 h, 16 h, 2, 3, 5, 7, and 10 days, and transferred into metal-free centrifuge tubes. Optical density (OD 600 nm) and pH were immediately measured from subsamples (5 mL) using, respectively a spectrophotometer (UV-1800, Shimadzu) and a pH-meter (NeoMet pH-200L), which was calibrated using three standard buffer solutions (pH 4, 7, and 10 at 27°C). Sediment/water mix samples were centrifuged (15 min, 8,000 rpm) and the supernatants were filtered on 0.2-μm cellulose acetate filters using pre-cleaned Minisart syringes, transferred to pre-cleaned FEP bottles and stored in closed plastic bags at 4°C until total dissolved TE analyses. Sediment pellets (0.5–1 g), collected at 3, 5, 7, and 10 days, were stored at −20°C for DNA extraction (in parallel with initial SED samples). The S/L ratio was maintained relatively constant during experiments. Forty milliliters of water and 0.5–1 g of sediment were collected simultaneously at each of the 9 sampling times from a 2-L water volume and a 32.8-g sediment mass, respectively, the initial and final S/L ratios displaying a variation of only ~2%.

### Analysis of Trace Elements

All samples were processed and analyzed in a trace metal clean HEPA filtered laboratory (ISO 7), using high purity acids (Fisher, Optima grade) and MilliQ water (*R* = 18.2 MΩ.cm^−1^). Both sets of PFA beakers and micropipette tips were cleaned in HCl (10%, 100°C, 24 h), rinsed and dried in a laminar flow cabinet (ISO 4). Sediment samples were leached with 9 mL of pure acid mixture (HF/HCl/HNO_3_, 1:6:2) and heated on a hot-block (120°C, 24 h). Solutions obtained were evaporated when almost dry and residues were dissolved in 100 mL of HNO_3_ (2%) prior to analysis. Water samples (SW and AWW) were diluted 1/20 in 2 % HNO_3_ before analysis. Concentrations of TE (As, Cd, U and V) in all samples were then evaluated using Inductively Coupled High Resolution Plasma Mass Spectrometry (HR-ICP-MS, Element XR, Thermo Scientific). To correct instrumental drift and possible matrix effects, internal standard elements (In) were added to the samples. Analytical results were validated using Certified Reference Material (MESS-4 for sediments and SLEW-3 for waters).

### DNA Extraction, PCR, and Sequencing Analyses of 16S rRNA Gene Fragments

DNA extraction from duplicated initial SED and sediment pellets of biotic conditions S and P (prepared in triplicates and collected at different times, as described above) was carried out using UltraClean Soil DNA Isolation Kit (MoBio Laboratories, Inc., CA), as previously described by Quéméneur et al. (2016). DNA was extracted from duplicated AWW and SW filters using PowerWater DNA Isolation Kit Sample (MO BIO Laboratories, Inc., CA). DNA was quantified using the Thermo Scientific Nano Drop 2000 spectrophotometer.

Bacterial abundance in collected samples was evaluated by real-time quantitative PCR of 16S rRNA genes using the 331F and 797R primers, as previously described by (Abdallah et al., 2016).

Bacterial and archaeal 16S rRNA genes were amplified by PCR using the 341F/815R prokaryotic universal primer set, as previously described by Dowd et al. (2008), and were sequenced by the MiSeq Illumina (paired-end 2 x 300 bp) platform of the Molecular Research Laboratory (Texas, USA). Raw sequences were analyzed using QIIME 1.9.1 as described by Caporaso et al. (2010). Briefly, the raw reads were checked for adapter, chimera and low-quality sequences. The trimmed reads were clustered into operational taxonomic units (OTU) using a 97% sequence identity threshold with UCLUST (Edgar, 2010). The taxonomic assignment was performed by UCLUST taxonomy. Low abundance OTU (< 0.005%) were filtered as recommended by Bokulich et al. (2013) and the OTU table was normalized by random subsampling to the smallest number of sequences (i.e., 18614). Similarity search by BLAST algorithm (Altschul et al., 1990) against the NCBI non-redundant (NR) reference database was performed for OTU representative sequences. The 16S rRNA gene sequences have been deposited in the Genbank database under the accession numbers MH002252-MH002311.

### Numerical and Statistical Analyses

The alpha diversity was calculated in QIIME using the Shannon (Shannon and Weaver, 1949) and Simpson (Simpson, 1949) indices. The beta diversity (Bray–Curtis similarity) metrics were calculated and a dendrogram was generated with *hclust* function in R to group samples into clusters. The Good's coverage was calculated according to the equation: C = 1–(n/N) where n is the number of OTU and N is the total number of sequences (Good, 1953). The relative OTU table was transformed by logarithm to down weight the influence of more abundant species masking shifts among less abundant species (Cabrol et al., 2012). The dynamics of microbial community structure along time were analyzed by Principal Coordinate Analysis (PCoA), using *pcoa* function of the *ape* package in R software (version 3.5.0), from the log-transformed OTU table. The temporal succession was visualized by a bubble plot on the PCoA representation, in which the symbol size was proportional to the elapsed time. Ellipses were drawn based on the standard deviation of points in groups defined by the presence/absence of acid effluent addition (*ordiellipse* function, confidence limit 0.9) and the significance of group separation according to acid effluent addition was tested by non-parametric (permutational) analysis of variance using distance matrix (Bray Curtis) with the *adonis* function (*p*-value 0.002). The first 50 OTU with highest variance were identified on the score/species biplot representation (the magnitude of OTU abundance change being proportional to the species arrow length). Their significant correlation with the ordination scores was tested by the *envfit* function (vegan package, R). The most discriminant OTU (i.e., with *p*-value < 0.001) explaining the sample distribution on the PCoA ordination were selected (yielding 32 species) and represented on the biplot. Collinearity between abiotic variables was tested by computing the pairwise Pearson correlation matrix between 8 variables (Table S2). “Time” and “AWW” variables were removed due to their high correlation (|ρ| > 0.9) with, respectively, dissolved Cd concentration and pH and U concentration. The linear correlation between the PCoA ordination of the microbial communities and key environmental parameters (after normalization) was investigated using the *envfit* function of the *vegan* library. Fitted environmental vectors were represented on the PCoA by arrows pointing to the direction of the increasing gradient and of which the length is proportional to the correlation coefficient between the variable and the ordination. The correlation significance of each variable was assessed by permutation tests. To explore the concerted effect of the 6 abiotic variables on the multivariate pattern of the microbial community, the *bioenv* routine (*vegan* package) was applied to test the 5 different possible models (i.e., subsets of environmental variables). The model providing the maximum rank correlation (Spearman coefficient) between a subset of environmental variables (Euclidian distances) and community dissimilarities (Bray Curtis distances) was identified. The significance of these correlations was tested by Mantel tests (*mantel* function).

The Mann-Whitney non-parametric test (U-test) was used to compare, two-by-two, the four different treatments (SA, S, P, PA) for each of these parameters: OD, pH, cadmium, uranium, arsenic, vanadium, diversity indices, and bacterial abundance. The U-test was also used to compare, for each parameter, the biotic (S and P) vs. abiotic (SA, PA) treatments, with effluent (P, PA) vs. without effluent (S, SA) treatments, as well as T0 vs. Tf conditions. The U-test was chosen for these comparisons because most of the treatments displayed a non-normal distribution according to several normality tests (Shapiro-Wilk, Anderson-Darling, Lilliefors tests). To find relationships between relative abundance of selected OTU and TE, we performed the Spearman correlation test (the non-parametric version of the Pearson correlation test) and we accepted correlation coefficients with *p*-values of < 0.05 as significant associations. Normality tests, the U-test, correlation tests and heatmap were performed with XLSTAT 2013.5.01 (Microsoft Excel add-in program). The heatmap displays the taxonomic affiliation obtained by the BLAST algorithm against the NCBI NR reference database.

## Results

### Physicochemical Characteristics of Initial Sediment, Seawater, and Acid Effluent Samples

The studied coastal surface sediment (SED) was mainly composed of sand (silt and clay were absent). Within the sand, medium (250–500 μm) and coarse (500–1000 μm) fractions were the majority (60 and 37%, respectively), while very fine (63–125 μm), fine (125–250 μm), and very coarse (1000–2000 μm) fractions represented together only 3% of the sand. SED contained low total organic carbon content (1.5% of the total sediment weight) and was characterized by a pH value of 6.3. It exhibited a multi-contamination involving various TE: 3.2 mg kg^−1^ for As, 16.4 mg kg^−1^ for Cd, 19.7 mg kg^−1^ for U, and 25.9 mg kg^−1^ for V. The main physicochemical properties of the initial SED used in resuspension experiments are summarized in Table 2 and the characteristics of the initial seawater (pH 8.3, SW) and acidic wastewater (pH 2.4, AWW) are given in Table S1.

**Table 2:** Chemical properties of sediment (SED) used in resuspension experiments and collected on the Sfax southern coast (Tunisia, South Mediterranean Sea).

	**SED**
pH *in situ*	6.3
Temperature *in situ* (°C)	31.3
Eh *in situ* (mV)	203
Water content (% of wet weight)	39.7
Total carbon (% of dry weight)	2.4
Organic carbon (% of dry weight)	1.4
Total nitrogen (% of dry weight)	0.2
Total sulfur (% of dry weight)	0.9
Total hydrogen (% of dry weight)	0.7
P (mg kg^−1^)	367–15110 (2456)^a^
**Trace element concentrations (mg.kg**^**−1**^**)**	
As	3.2
Cd	16.4
Co	1.5
Cr	330.8
Mn	113.5
Mo	8.1
Ni	34.9
Pb	0.2
U	19.7
V	25.9

a *Data obtained from Naifar et al. (2018)*.

### 16S rRNA Gene Diversity in Initial Sediment, Seawater, and Acid Effluent Samples

**Figure 2:**
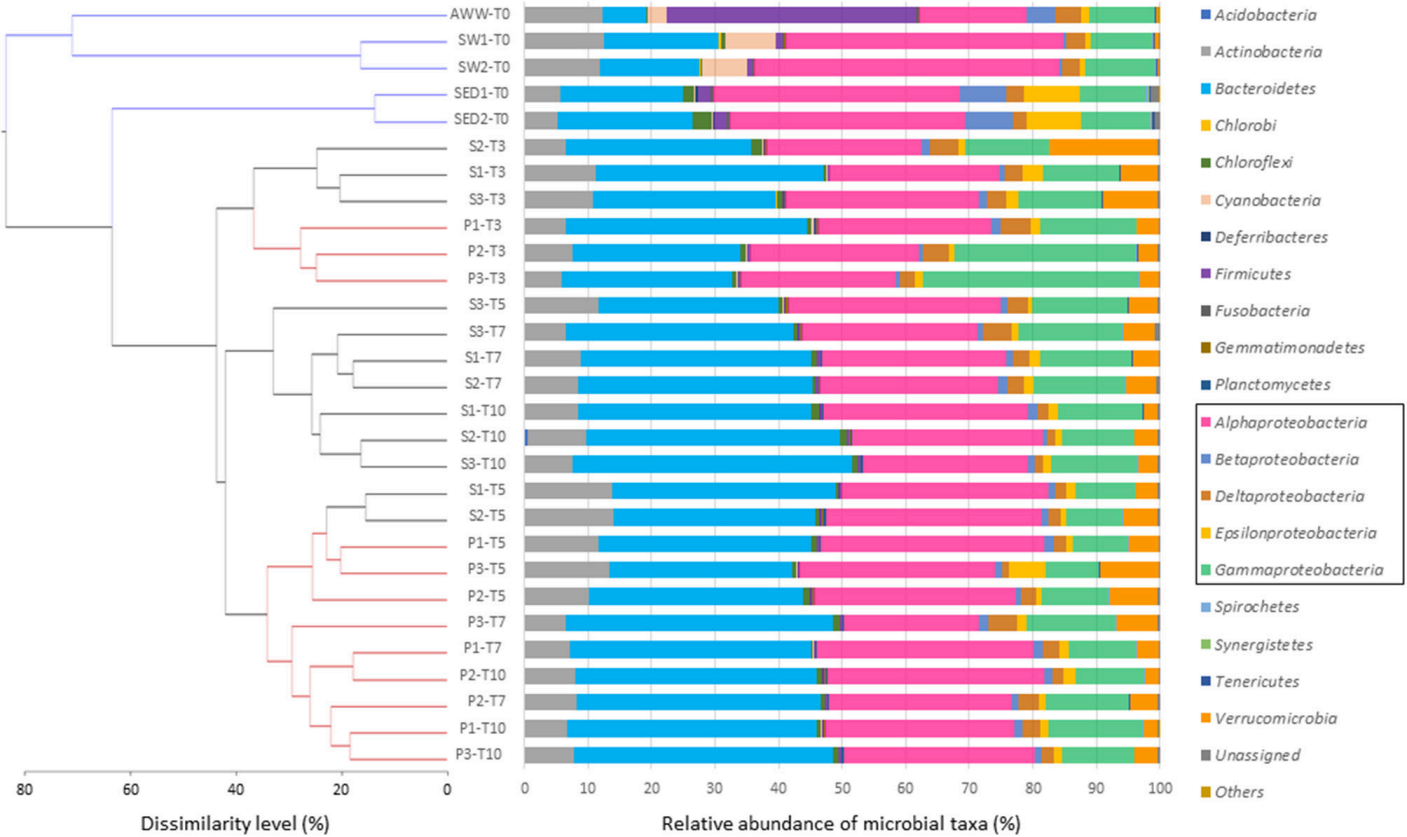
Composition of microbial communities at the phylum or class level in initial sediment (S), seawater (SW), and acid wastewater (AWW) of Sfax coast (south Mediterranean Sea), and in sediment samples collected from biotic incubations with or without AWW addition (S and P conditions, respectively) at 4 different times (T3, T5, T7, and T10 days). Each bar represents the color-coded relative abundance of microbial taxa in the studied samples. The dendrogram was constructed from the OTU abundance table using Bray–Curtis dissimilarity. The scale bar of the dendrogram represents the dissimilarity level (%) between microbial community.

The initial microbial diversity and bacterial abundance in sediment (SED), seawater (SW), and acidic wastewater (AWW) samples was estimated by 16S rRNA analyses based on next-generation sequencing (NGS) and quantitative PCR (qPCR). Considering sequence variability into replicate, the OTU numbers in SED, SW and AWW were 1041 ± 26, 1068 ± 26 and 651, respectively (Table S3). As expected from the drastic selective pressure existing in acidic and metal-rich effluent, both diversity indices and bacterial abundance were lower in AWW than in SED and SW. Initial community structures in SW and AWW were clearly separated, and most of them were segregated from the subsequent incubation communities (Figure 2). Field duplicate communities clustered together.

In the sediment, the microbial community was dominated by five bacterial phyla: *Proteobacteria* (67. 0 ± 1.0 %), *Bacteroidetes* (20.2 ± 1.0 %), *Actinobacteria* (5.4 ± 0.2 %), *Chloroflexi* (2.3 ± 0.7 %), *Firmicutes* (2.0 ± 0.0 %). Interestingly, the SED community was dominated by only 9 major OTU (>1 % of all sequences) belonging to 4 classes: *Alphaproteobacteria* (represented by *Thioclava* genus, >20 % of all sequences), *Epsilonproteobacteria* (*Sulfurovorum* and *Arcobacter* genera), *Betaproteobacteria* (*Azoarcus* genus), and *Flavobacteriia* (*Gaetbulibacter* and *Namhaeicola* genera) (Table S4). *Archaea* (*Methanobacteria* class) represented < 0.1% of the microbial community of SED.

In the seawater, the microbial community was dominated by four phyla (> 95 % of all sequences): *Proteobacteria* (60.4 ± 2.6 %), *Bacteroidetes* (16.8 ± 1.2 %), *Actinobacteria* (12.3 ± 0.3 %), and *Cyanobacteria* (7.5 ± 0.5 %) specific to SW. The SW was dominated by 14 OTU (>1% of all sequences): 9 alphaproteobacterial OTU related to *Rhodobacterales* genera, 2 cyanobacterial OTU, 3 actinobacterial, and 1 *Bacteroidetes* OTU (Table S5).

Despite its drastic pH and high TE concentrations, the AWW has a relatively high diversity (compared to acid mine drainage). The distribution of OTU strongly differs from SW and SED, mainly due to the dominance of *Firmicutes* (39.3 %), followed by *Proteobacteria* (37.1 %), *Actinobacteria* (12.2 %), *Bacteroidetes* (6.8 %), and *Cyanobacteria* (3.1 %). Among the 13 dominant OTU, 6 OTU were affiliated to *Firmicutes* and assigned to *Alicyclobacillaceae* (18.4 %), *Clostridiaceae* (3.4 %) and *Peptostreptococcaceae* (7.2 %) (Table S6).

### Variations of Optical Density and pH in Sediment Resuspension Experiments

**Figure 3:**
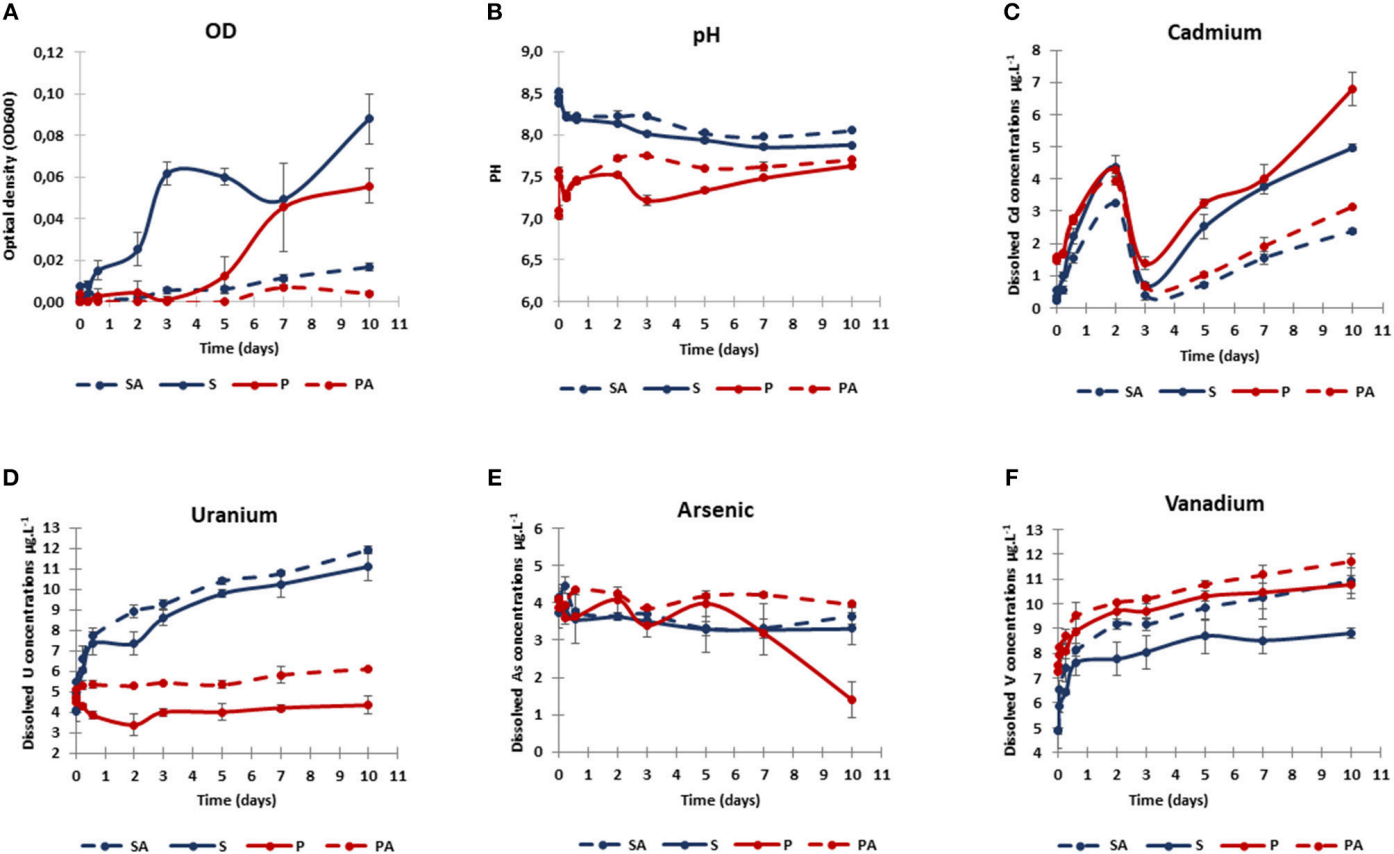
Dynamics of bacterial growth (optical density OD 600 nm; **A**), pH **(B)**, and dissolved concentrations of cadmium **(C)**, uranium **(D)**, arsenic **(E)**, and vanadium **(F)** during sediment resuspension experiments. Two treatments were tested: biotic sediment (S, blue), and biotic sediment with AWW addition (P, red). Corresponding abiotic controls (SA and PA) were represented by dotted lines. Values are means of triplicate resuspension microcosms ± standard deviations (error bars).

The evolution of optical density (OD) and pH in water during the 10-day sediment resuspension experiments are given in Figures 3A,B.

Significant OD increases were observed in the biotic conditions (S and P) between the beginning (T0) and the end (Tf) of the experiment (U-test, *p* < 0.01, *n* = 12), and compared to abiotic controls (SA, PA) for the whole experiment (U-test, *p* < 0.01, *n* = 108). Without AWW addition (S), the OD values increased rapidly up to 0.09 ± 0.01 after 10 days, with a relative maximum (0.06 ± 0.01) after 3 days. With AWW addition (P), the OD values increased more slowly up to 0.06 ± 0.01 after 10 days, suggesting that AWW increased lag times. Even though a slight OD increase was found in the abiotic controls between the beginning (T0) and the end (Tf) of the experiment (U-test, *p* < 0.05, *n* = 12), the much higher OD values recorded in biotic conditions suggest the occurrence of microbial growth in these latter conditions only (Figure 3A).

Without AWW addition (initial pH ~8.5), a pH decrease was observed during the 6 first hours (pH 8.2), which is probably due to the mixing of SW and SED. Average pH values continued to decrease until the tenth day (S: 7.9; SA: 8.1; Figure 3B). With AWW addition (initial pH 7.0), a pH increase was observed during the first hour (pH 7.5), which is probably due to the buffering capacity of the SED and SW. This increase was followed by a decrease (pH 7.2) during the next 6 h and by a divergence between P and PA. For the whole experiment, pH values were significantly lower with AWW addition in comparison to values without AWW addition (U-test, *p* < 0.001, *n* = 108; Figure 3B). No statistically significant difference in pH was observed between biotic and abiotic conditions from T0 to Tf (U-test, *p* > 0.05, *n* = 108), but pH values were significantly lower in the biotic conditions (S and P) than in the abiotic controls (SA and PA) if we only consider samples collected from 2 days of experiments (i.e., 2, 3, 5, 7, and 10 days; U-test, *p* < 0.01, *n* = 60; Figure 3B). With AWW addition, pH values were significantly lower in the biotic conditions (P) than in the abiotic controls (PA) over time (from T0 to Tf; U-test, *p* < 0.01, *n* = 54). The pH variations observed in the biotic conditions (S and P) suggest that the biogeochemical reactions controlled by bacteria induced decrease in pH.

### Metal Dynamics in Sediment Resuspension Experiments

Changes in dissolved arsenic (As), cadmium (Cd), uranium (U), and vanadium (V) concentrations were observed during 10-day remobilization experiments (Figures 3C–F).

At T0 when mixing SED with SW, an initial Cd release from SED was observed in the P conditions (1.46 ± 0.05 μg L^−1^), significantly stronger than in S conditions (0.21 ± 0.03 μg L^−1^; U-test, *p* < 0.01, *n* = 12). This may be explained by the initial Cd input resulting from AWW addition (i.e., 0.9 μg L^−1^, corresponding to 61% of the initial Cd). Independently of AWW addition, Cd was strongly remobilized during the 2 first days, and then returned rapidly to initial low levels at 3 days, prior to a progressive increase until the end of the experiments. At Tf, significant Cd difference was observed between biotic and abiotic conditions (U-test, *p* < 0.05, *n* = 10). Cd release was twice higher in the biotic conditions (S: 4.98 ± 0.11 μg L^−1^; P: 6.79 ± 0.51 μg L^−1^) than in the corresponding abiotic conditions (Figure 3C), suggesting that microorganisms might play a role in the Cd remobilization.

The initial concentrations of dissolved U were similar in S and P conditions (4.1–4.6 μg L^−1^) (Figure 3D). With AWW addition, no significant U remobilization was observed between the biotic condition P at T0 and Tf (U-test, *p* > 0.05, *n* = 6), and a slight U increase, albeit insignificant, was detected between abiotic condition PA at T0 and Tf (5.81 ± 0.39 μg L^−1^ at 10 days; U-test, *p* > 0.05, *n* = 6). In contrast, U release was accentuated at pH 8.5, in which its concentration almost tripled after 10 days (SA: 12.42 ± 0.89 μg L^−1^; S: 11.10 ± 0.71 μg L^−1^), indicating that U mobilization increased in natural seawater (without AWW addition).

Initial dissolved As concentration was around 4 μg L^−1^ in all experiments (Figure 3E). Dissolved As levels remained stable until day 10 in all cases, except in biotic condition P, where it significantly decreased after 7 days, down to 1.41 ± 0.40 μg L^−1^ (T10; significantly lower As values for P compared to other conditions at Tf; U-test, *p* < 0.05, *n* = 11), suggesting biogenic As immobilization after AWW addition.

Initial dissolved V levels varied between P and S conditions, but dissolved V presented similar initial values under biotic or abiotic experiments (~4.9 μg L^−1^ for S and SA; ~7.4 μg L^−1^ for P and PA; Figure 3F). These results may be partly explained by the initial V input from AWW addition (i.e., 1.6 μg L^−1^, corresponding to 21% of the initial V). A gradual increase in dissolved V was observed in all experiments, especially during the first day and the remobilization slowed down until the 10th day. The final dissolved V tended to be lower in the biotic condition S (8.8 ± 0.2 μg L^−1^) than in other conditions (SA: 10.92 ± 0.72 μg L^−1^; P: 10.77 ± 0.34 μg L^−1^; PA: 11.71 ± 0.29 μg L^−1^; significantly lower V values for S compared to other conditions at Tf; U-test, *p* < 0.05, *n* = 11), indicating that V remobilization was biologically decreased in natural seawater (without AWW addition).

### Microbial Community Dynamics in the Resuspension Experiments

The dynamics of microbial diversity and bacterial abundance were monitored over time (0, 3, 5, 7, and 10 days), in the pelletized sediment suspensions from the triplicated biotic conditions using 16S rRNA NGS and qPCR analyses (P with AWW addition and S without AWW addition). After 3-day incubation, the number of observed OTU increased from 1041 ± 6 (for initial SED) to 1078 on average, in both S and P conditions independently of acidification. A gradual increase in OTU number was observed over time in S conditions (1113 ± 10 after 10 days; Table S3), while no difference in OTU richness and Shannon index was observed in P conditions. On the contrary, bacterial abundance displayed a significant decrease from 2.87 ± 0.96 x 10^9^ (for initial SED) to 1.77 ± 0.14 x 10^8^ and 2.50 ± 0.27 x 10^8^, respectively in both S and P experiments (after 10 days, U-test, *p* < 0.05; Table S3). No significant difference between S and P conditions was observed at Tf.

Change in the microbial community structure was shown in the hierarchical dendrogram based on Bray–Curtis similarity (Figure 2). All the incubation samples were separated from the initial samples. After 3-day incubation (T3), samples of conditions S and P clustered together and replicate samples from the same conditions (S1, S2, S3 or P1, P2, P3) grouped also together. With the sole exception of S3-T5 separated from S1-T5 and S2-T5, the dynamics and clustering of microbial communities were reproducible between samples triplicates over time. After 7 days and until the end of the incubation, replicate samples exposed to acidic effluent (P, in red) clustered together clearly apart from replicate samples without acidic effluent (S, in black).

On average, bacterial communities in the S and P conditions were mainly composed of the following phyla/classes: *Bacteroidetes* (35%), *Alphaproteobacteria* (29%), *Gammaproteobacteria* (14%)*, Actinobacteria* (9%), and *Verrucomicrobia* (5%; Figure 2). Compared to the initial SED community, *Alphaproteobacteria* decreased over time (from 37% initially to ~28% at the end), while *Bacteroidetes* were enriched in both S and P conditions (from 20% initially to ~35% at the end; Figure 2). No difference in low archaeal proportion (< 0.1% of microbial community) was observed depending on treatment and time.

### Correlations Between Microbial Communities and Resuspension Experimental Conditions

**Figure 4:**
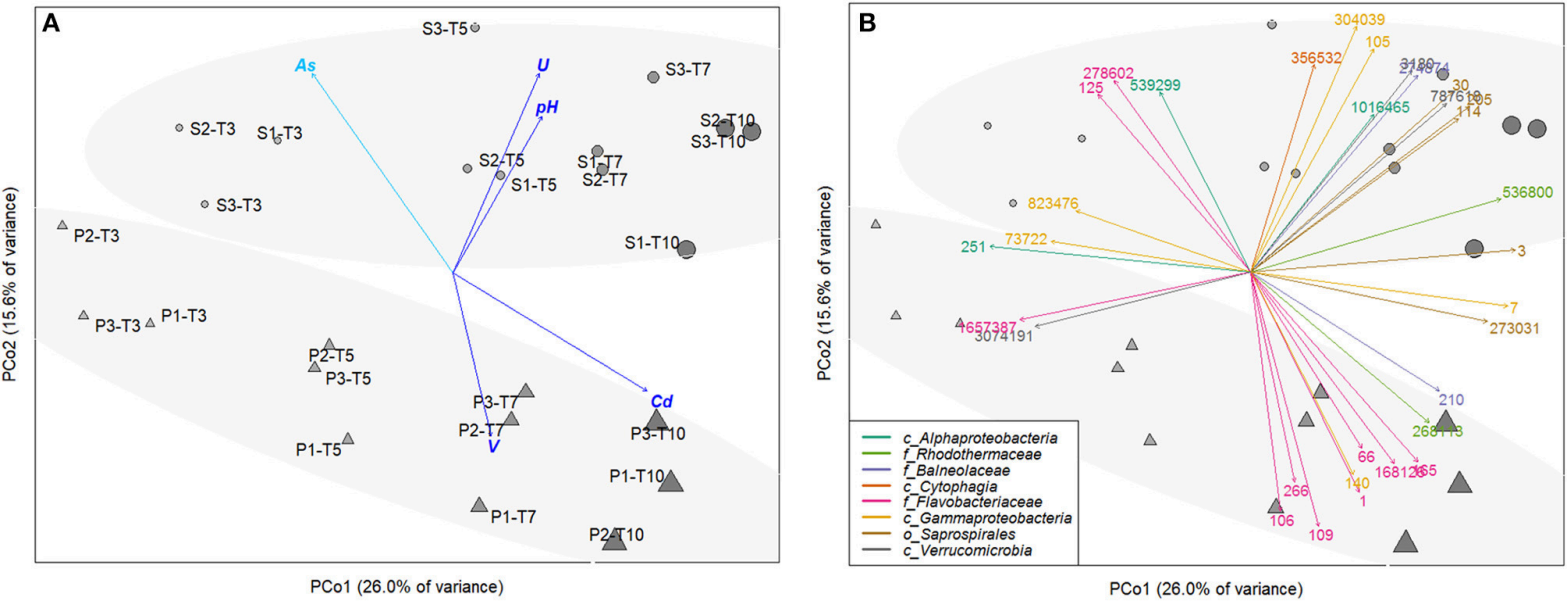
Principal Coordinate Analysis (PCoA) computed from the OTU abundance table of resuspended sediments, either exposed (P,▴) or not (S,∙) to acidic effluent (AWW), performed in triplicate (each replicate is indicated by the second digit, from 1 to 3), and collected at 4 different times along the incubation (time T3 to T10). The size and color intensity of symbols is proportional to elapsed time. Ellipses represent sample partitioning (Bray Curtis dissimilarity) between the P and S groups (standard deviation at 90% confidence, *p*-value 0.002). **(A)** Correlation between ordination and unrelated environmental variables (after removing the linearly-correlated variables). Only significant correlations are represented (*p*-value < 0.01, in light blue, *p*-value < 0.001, in dark blue). **(B)** Identification of 32 most discriminant OTU (labeled with their OTU number). Arrow colors represent the OTU affiliation at different taxonomic levels (class/order/family, represented by c/o/f respectively). BLAST affiliation of those OTU can be found in Table S8.

**Figure 5:**
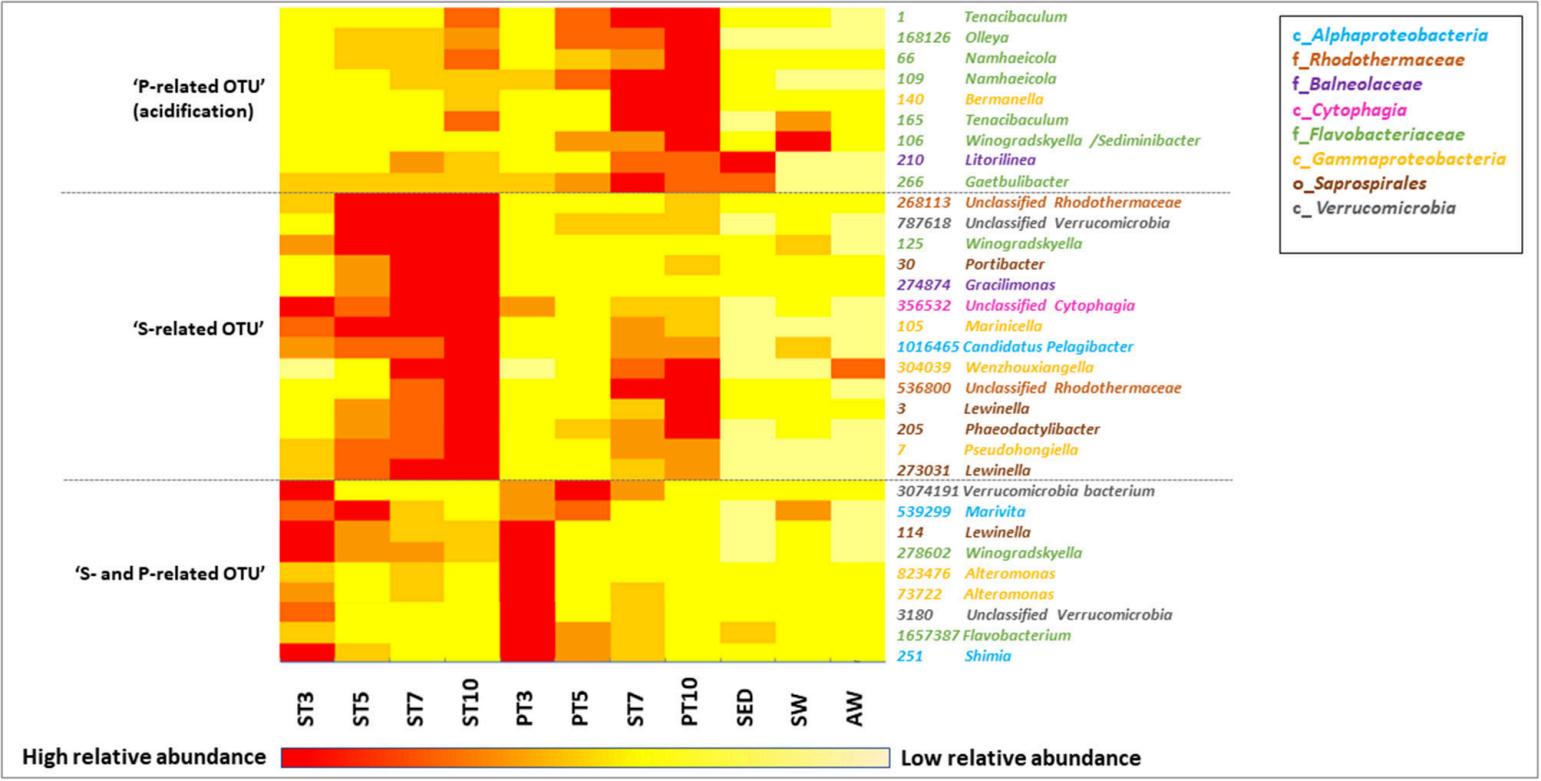
Heat map showing the relative abundance of the most discriminant OTU selected from the PCoA of initial samples (SED, SW, and AWW) and samples collected during resuspension experiments (at 4 different times from T3 to T10), either in exposed (P) or not (S) to AWW. Data are average abundances calculated on biological triplicates. The color intensity for each panel corresponds to the OTU abundance, red indicates high level of relative abundance, while yellow indicates low relative abundance.

Principal Coordinate Analysis (PCoA) based on the relative OTU abundances showed a clear temporal succession along the PC1-axis (r2 = 0.84, *p* < 0.001 for time effect), evidencing the adaptation of microbial communities to resuspension conditions over time (Figure 4A). After 3 day-incubation, microbial communities were still highly similar in S and P conditions, with 10 common discriminate OTU affiliated to *Alphaproteobacteria* and *Gammaproteobacteria* classes, *Flavobacteriaceae* family, *Verrucomicrobia* class (Figures 4B, 5). From 5-day incubation, the PCoA highlighted a clear separation of microbial communities into two groups along the PC2-axis according to the presence/absence of AWW, which was confirmed by PERMANOVA (r2 = 0.76, *p* < 0.01). The divergence increased with time and the AWW addition was the most important driving force of the microbial community structure. Higher pH and higher diversity were significantly correlated with S microbial communities (r2 = 0.59, *p* < 0.01 and r2 = 0.49, *p* < 0.01, respectively). Dissolved U levels were also significantly correlated with S communities (r2 = 0.88, *p* < 0.01), especially at intermediary times of the incubations (Figure 4A), suggesting enhanced U immobilization in presence of acidic effluent. However, the similar trend between biotic and abiotic U kinetics (Figure 3D) suggests an effect of U as driver of the microbial structure rather than a potential role of microbial communities in U mobilization. On the other hand, the significant and high linear correlation between dissolved Cd levels and microbial communities exposed to acidic effluent (r2 = 0.87, *p* < 0.01), especially at the end (Figures 4A, 3C), fitted with our physicochemical kinetics, suggesting that microorganisms may enhance Cd remobilization. The same microbial effect was observed, to a lower extent, on V remobilization, especially at intermediate times of the incubation (r2 = 0.54, *p* < 0.05). When tested altogether, the concerted effect of the abiotic variables on the multivariate pattern of the microbial community revealed that a simple model including only 2 variables (namely pH and Cd concentration) best explained the community structure (r^2^ = 0.6265, *p* < 0.01). Models including more variables (As, U, V) were also significant (*p* < 0.01) but less strongly correlated to the community structure (r2 = 0.49 to 0.61; Table S7).

### Dynamics of Representative OTU in the Sediment Resuspension Experiments

The taxonomic affiliation of the 32 representative OTU selected from the PCoA is given in Table S8. The relative abundance of these OTU over time was presented in the heatmap (Figure 5), which separated them into 3 groups according to their enrichment in S or P conditions. The relative abundance and diversity of the *Bacteroidetes* OTU differed between S and P conditions. The first group (named “P-related OTU”) contained the majority of the representative OTU (9) explaining the separation of P communities at the end of the experiment. They were mainly affiliated to the *Flavobacteriaceae* family within the *Bacteroidetes* phylum (1, 66, 106, 109, 165, 168126; Figure 4B, Table S6), suggesting a competitive advantage of these marine bacteria under acidified P conditions. The second group (named “S-related OTU”) contained 15 OTU, which seem to be more abundant in S than P condition, but no statistically significant difference was observed between conditions. Eight of them were mainly related to *Bacteroidetes*. Five *Saprospirales* OTU (3, 30, 114, 205, 273031) were enriched at Tf in S conditions, suggesting they might be adapted to sediment resuspension (Figure 5). The third group (named “S- and P-related OTU”) includes the 10 common OTU observed after 3 days, which decline over time, suggesting they were not adapted to the conditions imposed by sediment resuspension.

Spearman's rank correlation analysis showed significant positive correlations (r_s_ > 0.70, *p*-value < 0.05) between dissolved Cd levels and relative abundance of several OTU (e.g., 3, 7, 66, 109, 165, 140, 210, 168126; Table S9). The affiliation of these OTU at the genus level (when available) is provided on the heatmap (Figure 5) and Table S8. For example, relative abundance of *Flavobacteriaceae* OTU 165, affiliated to *Tenacibaculum* genus, increased by 4 times over time and were highly correlated with Cd levels (r_s_ = 91 respectively; Table S7). Relative abundance of four *Bacteroidetes* OTU positively correlated with two TE: (i) Cd and V, such as *Flavobacteriaceae* OTU 109 and 168126 (*Namhaeicola* and *Olleya*, respectively) and *Rhodothermaceae* OTU 536800, or (ii) Cd and U that correlated with *Saprospirales* OTU 3 (*Lewinella*), suggesting multi-resistance to metals. No significant correlation was observed between selected OTU and As.

## Discussion

The coastal marine ecosystems of the Gulf of Gabès (GG, southern Mediterranean Sea) are impacted by long-term discharges from fertilizer industry waste containing trace metals (El Zrelli et al., 2017; El Kateb et al., 2018). Our results demonstrated that resuspension of surface contaminated sediment from the Sfax coast led to high releases of Cd, U and V in seawater. High concentrations of Cd were initially found in surface sediment collected in front of mixed wastewater discharge into the seawater (El Hakmouni Wadi), as detected in previous studies on surface sediments of the Sfax southern coast (Zouch et al., 2017; Naifar et al., 2018). The fate and distribution of As, Cd, U and V are driven by complex processes controlled by abiotic and biotic parameters. Cd remobilization was biologically enhanced in our experiments, while remobilization of U from the sediment seemed to be mainly enhanced by abiotic factors, such as pH or oxygenation (Moon et al., 2007). Biotic factors are known to control both U and V immobilization, but in anoxic sediments, in which they can be bioreduced or trapped by hydrogen sulfide bioproduced from OM (Cumberland et al., 2016; Reijonen et al., 2016). In our experiments conducted under aerobic conditions, arsenic (As) was efficiently immobilized by the surface oxic sediments. Indeed, As is known to be less mobile under aerobic conditions than under anaerobic conditions in reducing sediment porewater (Bataillard et al., 2014). Moreover, a previous resuspension study of estuarine sediments showed that As was released ten times less from a surface oxic layer (the 0–0.5 cm) than from a deep anoxic layer sediment (13–15 cm depth; Saulnier and Mucci, 2000).

Acidic industrial effluent induced an increase in the content of several trace metals (e.g., Cd, V) in our resuspension experiment and could influence metal dynamics by modifying some physico-chemical parameters, especially pH, which control TE chemical form and mobility (Kiratli and Ergin, 1996; Eggleton and Thomas, 2004; Millero et al., 2009; Wang Z. et al., 2015). Martín-Torre et al. (2013) have shown that metal response to pH variation varies from one element to another. According to our study, Cd was not controlled by neutral/marine pH (7–8.5) range, because it forms strong chloro complexes in seawater, which are weakly influenced by pH change (Millero et al., 2009; Martín-Torre et al., 2013; Bruland, 2014). U mobility was controlled by pH (immobilized at initial pH < 8 and mobilized at initial pH > 8), because U is known to form strong complexes with carbonates and hydroxides influenced by pH (Millero et al., 2009; Cumberland et al., 2016). V release was observed in all experimental conditions, according to previous studies reporting high V mobility at neutral/marine pH (Brunori et al., 2005; Reijonen et al., 2016). In contrast, As was not remobilized during our experiment conducted at neutral/marine pH, in agreement with previous studies showing low As solubilization from contaminated marine sediment and processing waste under aerobic and neutral pH conditions (Al-Abed et al., 2007; Martín-Torre et al., 2013). In the neutral/marine pH region, the low As release is probably due to the co-sorption of As and Fe in sediments (Saulnier and Mucci, 2000; Al-Abed et al., 2007).

Acidic wastewater addition impacted bacterial community dynamics in our sediment resuspension. Lower bacterial diversity and changes in bacterial composition were observed in samples with acidic effluent addition, suggesting that slight pH decrease and/or TE input (e.g., Cd), was selective pressure for some sensitive marine bacteria. A shift within *Bacteroidetes* phylum diversity was observed depending on AWW addition. Indeed, the relative abundance of *Flavobacteriaceae* species increased with addition of AWW containing trace metals and leading to a pH decrease. *Flavobacteriaceae* phylotypes (e.g., *Gaetbulibacter, Tenacibaculum*, and *Winogradskyella*) were dominant in the initial samples and were linked to Cd and V levels in our incubations. Some of them were already reported to be positively correlated with Cd levels in a previous study reporting the effect of Cd (up to 100 μg L^−1^) on the microbial community of the East China Sea (Wang K. et al., 2015). The pH is also a key factor influencing bacterial community structure, as recently reported by several studies dealing with marine sediment, contaminated soil and freshwater ecosystems (Liu et al., 2015; Currie et al., 2017; Wu et al., 2017). As observed in our study, Krause et al. (2012) reported that slight acidification (small changes in pH from 8.2 to 7.7), due to the rise of anthropogenic CO_2_ emissions, cause shifts in bacterial communities in the North Sea, and they identified many pH-sensitive groups (e.g., *Flavobacteriaceae*). Consequently, the acidification and contamination of south Mediterranean coastal areas by phosphate fertilizer industrial discharge may affect marine ecological processes driven by bacteria, especially the phylum *Bacteroidetes* occurring in the coastal sediment and seawater, which are essential for the organic matter mineralization (Cottrell and Kirchman, 2000; O'Sullivan et al., 2006).

In conclusion, the input of acidic wastewater affected both microbial diversity and trace metal dynamics during resuspension of contaminated sediments. The effect of AWW addition was metal-dependant and mainly visible on the diversity of the phylum *Bacteroidetes*, which is assumed to be important in OM degradation. Among *Bacteroidetes*, members of *Flavobacteriaceae* seem to be well adapted to the acidification and metallic pollution. Although other parameters such as nutrients or organic contaminants (e.g., hydrocarbons) may also contribute to the dynamics of both metals and microbial communities in our incubations, our results show for the first time the effect of an extremely acidic effluent on coastal microbial communities and give some interesting insights concerning the fate of metals during resuspension events, which could be useful as bioindicators of environmental impacts or to predict potential metal contamination in coastal marine ecosystems.

## Author Contributions

LC, FK, HZo, SC, MQ, and MT designed experiments. FK, HZa, HZo, and MQ collected samples. HZo and MQ performed experiments. HZo performed microbial diversity analyses, SC and HZo performed metal analyses, LC, MT, and HZo performed numerical and statistical analyses. HZo wrote the manuscript in collaboration with MQ. All authors read and commented on the draft manuscript. All authors agreed to the final version.

### Conflict of Interest Statement

The authors declare that the research was conducted in the absence of any commercial or financial relationships that could be construed as a potential conflict of interest.

## References

[B1] Abdallah B. M. Karray F. Mhiri N. Mei N. Quéméneur M. Cayol J. L.. (2016). Prokaryotic diversity in a Tunisian hypersaline lake, Chott El Jerid. Extremophiles 20, 125–138. 10.1007/s00792-015-0805-7 26724953

[B2] Al-Abed S. R. Jegadeesan G. Purandare J. Allen D. (2007). Arsenic release from iron rich mineral processing waste: Influence of pH and redox potential. Chemosphere 66, 775–782. 10.1016/j.chemosphere.2006.07.045 16949129

[B3] Altschul S. F. Gish W. Miller W. Myers E. W. Lipman D. J. (1990). Basic local alignment search tool. J. Mol. Biol. 215, 403–410. 10.1016/S0022-2836(05)80360-2 2231712

[B4] Ayadi N. Aloulou F. Bouzid J. (2015). Assessment of contaminated sediment by phosphate fertilizer industrial waste using pollution indices and statistical techniques in the Gulf of Gabes (Tunisia). Arab. J. Geosci. 8, 1755–1767. 10.1007/s12517-014-1291-4

[B5] Ayata S. D. Irisson J. O. Aubert A. Berline L. Dutay J. C. Mayot N. (2018). Regionalisation of the Mediterranean basin, a MERMEX synthesis. Prog. Oceanogr. 163, 7–20. 10.1016/j.pocean.2017.09.016

[B6] Bataillard P. Grangeon S. Quinn P. Mosselmans F. Lahfid A. Wille G. (2014). Iron and arsenic speciation in marine sediments undergoing a resuspension event: the impact of biotic activity. J. Soils Sediments 14, 615–629. 10.1007/s11368-013-0829-5

[B7] Ben Salem Z. Ayadi H. (2016). Heavy metal accumulation in *Diplodus annularis, Liza aurata*, and *Solea vulgaris* relevant to their concentration in water and sediment from the southwestern Mediterranean (coast of Sfax). Environ. Sci. Pollut. Res. 23, 13895–13906. 10.1007/s11356-016-6531-6 27040537

[B8] Bokulich N. A. Subramanian S. Faith J. J. Gevers D. Gordon I. Knight R.. (2013). Quality-filtering vastly improves diversity estimates from Illumina amplicon sequencing. Nat. Methods 10, 57–59. 10.1038/nmeth.2276 23202435 PMC3531572

[B9] Bruland K. W. (2014). Complexation of cadmium by natural in the organic ligands central North Pacific. Limnol. Oceanogr. 37, 1008–1017. 10.4319/lo.1992.37.5.1008

[B10] Brunori C. Cremisini C. Massanisso P. Pinto V. Torricelli L. (2005). Reuse of a treated red mud bauxite waste: Studies on environmental compatibility. J. Hazard. Mater. 117, 55–63. 10.1016/j.jhazmat.2004.09.010 15621353

[B11] Cabrol L. Malhautier L. Poly F. Lepeuple A. S. Fanlo J. L. (2012). Bacterial dynamics in steady-state biofilters: beyond functional stability. FEMS Microbiol. Ecol. 79, 260–271. 10.1111/j.1574-6941.2011.01213.x 22029727

[B12] Cabrol L. Quéméneur M. Misson B. (2017). Inhibitory effects of sodium azide on microbial growth in experimental resuspension of marine sediment. J. Microbiol. Methods 133, 62–65. 10.1016/j.mimet.2016.12.021 28039035

[B13] Cantwell M. G. Burgess R. M. (2004). Variability of parameters measured during the resuspension of sediments with a particle entrainment simulator. Chemosphere 56, 51–58. 10.1016/j.chemosphere.2004.01.033 15109879

[B14] Caporaso J. G. Kuczynski J. Stombaugh J. Bittinger K. Bushman F. D. Costello E. K.. (2010). QIIME allows analysis of high- throughput community sequencing data. Nat. Met. 7, 335–336. 10.1038/nmeth.f.303 20383131 PMC3156573

[B15] Cichy B. Jaroszek H. Paszek A. (2014). Cadmium in phosphate fertilizers; ecological and economical aspects. Chemik 68, 20–22.

[B16] Cottrell M. T. Kirchman D. L. (2000). Natural assemblages of marine proteobacteria adn members of the *Cytophaga-Flavobacter* clustler consuming low- and high- molecular-weight dissolved organic matter. Appl. Environ. Microbiol. 66, 1692–1697. 10.1128/AEM.66.4.1692-1697.2000 10742262 PMC92043

[B17] Cumberland S. A. Douglas G. Grice K. Moreau J. W. (2016). Uranium mobility in organic matter-rich sediments: a review of geological and geochemical processes. Earth-Science Rev. 159, 160–185. 10.1016/j.earscirev.2016.05.010

[B18] Currie A. R. Tait K. Parry H. de Francisco-Mora B. Hicks N. Mark Osborn A.. (2017). Marine microbial gene abundance and community composition in response to ocean acidification and elevated temperature in two contrasting coastal marine sediments. Front. Microbiol. 8:1599. 10.3389/fmicb.2017.01599 28878754 PMC5572232

[B19] Dang D. H. Lenoble V. Durrieu G. Omanović D. Mullot J. U. Mounier S.. (2015). Seasonal variations of coastal sedimentary trace metals cycling: Insight on the effect of manganese and iron (oxy)hydroxides, sulphide and organic matter. Mar. Pollut. Bull. 92, 113–124. 10.1016/j.marpolbul.2014.12.048 25596891

[B20] Dowd S. E. Callaway T. R. Wolcott R. D. Sun Y. McKeehan T. Hagevoort R. G.. (2008). Evaluation of the bacterial diversity in the feces of cattle using 16S rDNA bacterial tag-encoded FLX amplicon pyrosequencing (bTEFAP). BMC Microbiol. 8, 1–8. 10.1186/1471-2180-8-125 18652685 PMC2515157

[B21] Edgar R. C. (2010). Search and clustering orders of magnitude faster than BLAST. Bioinformatics 26, 2460–2461. 10.1093/bioinformatics/btq461 20709691

[B22] Eggleton J. Thomas K. V. (2004). A review of factors affecting the release and bioavailability of contaminants during sediment disturbance events. Environ. Int. 30, 973–980. 10.1016/j.envint.2004.03.001 15196845

[B23] El Kateb A. Stalder C. Rüggeberg A. Neururer C. Spangenberg J. E. Spezzaferri S. (2018). Impact of industrial phosphate waste discharge on the marine environment in the Gulf of Gabes (Tunisia). PLoS ONE 13:e0197731. 10.1371/journal.pone.0197731 29771969 PMC5957445

[B24] El Zrelli R. Courjault-Radé P. Rabaoui L. Castet S. Michel S. Bejaoui N. (2015). Heavy metal contamination and ecological risk assessment in the surface sediments of the coastal area surrounding the industrial complex of Gabes city, Gulf of Gabes, SE Tunisia. Mar. Pollut. Bull. 101, 922–929. 10.1016/j.marpolbul.2015.10.047 26526855

[B25] El Zrelli R. Courjault-Radé P. Rabaoui L. Daghbouj N. Mansour L. Balti R.. (2017). Biomonitoring of coastal pollution in the Gulf of Gabes (SE, Tunisia): use of Posidonia oceanica seagrass as a bioindicator and its mat as an archive of coastal metallic contamination. Environ. Sci. Pollut. Res. 24, 22214–22225. 10.1007/s11356-017-9856-x 28795327

[B26] El Zrelli R. Rabaoui L. Ben Alaya M. Daghbouj N. Castet S. Besson P.. (2018). Seawater quality assessment and identification of pollution sources along the central coastal area of Gabes Gulf (SE Tunisia): evidence of industrial impact and implications for marine environment protection. Mar. Pollut. Bull. 127, 445–452. 10.1016/j.marpolbul.2017.12.012 29475683

[B27] Fonti V. Dell'Anno A. Beolchini F. (2013). Influence of biogeochemical interactions on metal bioleaching performance in contaminated marine sediment. Water Res. 47, 5139–5152. 10.1016/j.watres.2013.05.052 23866143

[B28] Fourati R. Tedetti M. Guigue C. Goutx M. Garcia N. Zaghden H. (2018a). Sources and spatial distribution of dissolved aliphatic and polycyclic aromatic hydrocarbons in surface coastal waters of the Gulf of Gabès (Tunisia, Southern Mediterranean Sea). Prog. Oceanogr. 163, 232–247. 10.1016/j.pocean.2017.02.001

[B29] Fourati R. Tedetti M. Guigue C. Goutx M. Zaghden H. Sayadi S.. (2018b). Natural and anthropogenic particulate-bound aliphatic and polycyclic aromatic hydrocarbons in surface waters of the Gulf of Gabès (Tunisia, southern Mediterranean Sea). Environ. Sci. Pollut. Res. 25, 2476–2494. 10.1007/s11356-017-0641-7 29127633

[B30] Gadd G. M. (2000). Bioremedial potential of microbial mechanisms. Curr Opin Biotechnol. 11, 271–279. 10.1016/S0958-1669(00)00095-1 10851150

[B31] Gadd G. M. (2004). Microbial influence on metal mobility and application for bioremediation. Geoderma 122, 109–119. 10.1016/j.geoderma.2004.01.002

[B32] Gadd G. M. (2010). Metals, minerals and microbes: Geomicrobiology and bioremediation. Microbiology 156, 609–643. 10.1099/mic.0.037143-0 20019082

[B33] Gargouri D. Azri C. Serbaji M. M. Jedoui Y. Montacer M. (2011). Heavy metal concentrations in the surface marine sediments of Sfax Coast, Tunisia. Environ. Monit. Assess. 175, 519–530. 10.1007/s10661-010-1548-7 20533086

[B34] Ghannem N. Gargouri D. Sarbeji M. M. Yaich C. Azri C. (2014). Metal contamination of surface sediments of the Sfax–Chebba coastal line, Tunisia. Environ. Earth Sci. 72, 3419–3427. 10.1007/s12665-014-3248-z

[B35] Ghilardi M. Psomiadis D. Cordier S. Delanghe-Sabatier D. Demory F. Hamidi F. (2012). The impact of early- to mid-Holocene palaeoenvironmental changes on Neolithic settlement at Nea Nikomideia, Thessaloniki plain, Greece. Quat. Int. 266, 47–61. 10.1016/j.quaint.2010.12.016

[B36] Gillan D. C. Danis B. Pernet P. Joly G. Dubois P. (2005). Structure of sediment-associated microbial communities along a heavy-metal contamination gradient in the marine environment. Appl. Environ. Microbiol. 71, 679–690. 10.1128/AEM.71.2.679-690.2005 15691917 PMC546797

[B37] Goni-Urriza M. Moussard H. Lafabrie C. Carré C. Bouvy M. Sakka Hlaili A. (2018). Consequences of contamination on the interactions between phytoplankton and bacterioplankton. Chemosphere 195, 212–222. 10.1016/j.chemosphere.2017.12.053 29268179

[B38] Good J. I. (1953). The population frequencies of species and the estimation of population parameters. Biometrika 40, 237–264. 10.1093/biomet/40.3-4.237

[B39] Guigue C. Tedetti M. Dang D. H. Mullot J.-U. Garnier C. Goutx M. (2017). Remobilization of polycyclic aromatic hydrocarbons and organic matter in seawater during sediment resuspension experiments from a polluted coastal environment: insights from Toulon Bay (France). Environ. Pollut. 229, 627–638. 10.1016/j.envpol.2017.06.090 28689151

[B40] Guzmán H. M. Jarvis K. E. (1996). Vanadium century record from Caribbean reef corals: a tracer of oil pollution in Panama. Ambio 25, 523–526.

[B41] Hamdi I. Denis M. Bellaaj-Zouari A. Khemakhem H. Bel Hassen M. Hamza A. (2015). The characterisation and summer distribution of ultraphytoplankton in the Gulf of Gabès (Eastern Mediterranean Sea, Tunisia) by using flow cytometry. Cont. Shelf Res. 93, 27–38. 10.1016/j.csr.2014.10.002

[B42] Hassen M. B. Hamza A. Drira Z. Zouari A. Akrout F. Messaoudi S. (2009). Phytoplankton-pigment signatures and their relationship to spring-summer stratification in the Gulf of Gabes. Estuar. Coast. Shelf Sci. 83, 296–306. 10.1016/j.ecss.2009.04.002

[B43] Jong T. Parry D. L. (2003). Removal of sulfate and heavy metals by sulfate reducing bacteria in short-term bench scale upflow anaerobic packed bed reactor runs. Water Res. 37, 3379–3389. 10.1016/S0043-1354(03)00165-9 12834731

[B44] Kalnejais L. H. Martin W. R. Bothner M. H. (2010). The release of dissolved nutrients and metals from coastal sediments due to resuspension. Mar. Chem. 121, 224–235. 10.1016/j.marchem.2010.05.002

[B45] Kim E. H. Mason R. P. Porter E. T. Soulen H. L. (2006). The impact of resuspension on sediment mercury dynamics, and methylmercury production and fate: a mesocosm study. Mar. Chem. 102, 300–315. 10.1016/j.marchem.2006.05.006

[B46] Kiratli N. Ergin M. (1996). Partitioning of heavy metals in surface Black Sea sediments. Appl. Geochemistry 11, 775–788. 10.1016/S0883-2927(96)00037-6

[B47] Krause E. Wichels A. Giménez L. Lunau M. Schilhabel M. B. Gerdts G. (2012). Small changes in pH have direct effects on marine bacterial community composition: a microcosm approach. PLoS ONE 7:e47035. 10.1371/journal.pone.0047035 23071704 PMC3469576

[B48] Liu S. Ren H. Shen L. Lou L. Tian G. Zheng P.. (2015). pH levels drive bacterial community structure in the Qiantang River as determined by 454 pyrosequencing. Front. Microbiol. 6:285. 10.3389/fmicb.2015.00285 25941515 PMC4403504

[B49] Martín-Torre M. C. Payán M. C. Galán B. Coz A. Viguri J. R. (2013). The use of leaching tests to assess metal release from contaminated marine sediment under CO2 leakages from CCS. Energy Procedia 51, 40–47. 10.1016/j.egypro.2014.07.005

[B50] Mayot N. D'Ortenzio F. D'Alcalà M. R. Lavigne H. Claustre H. (2016). Interannual variability of the Mediterranean trophic regimes from ocean color satellites. Biogeosciences 13, 1901–1917. 10.5194/bg-13-1901-2016

[B51] Millero F. Woosley R. DiTrolio B. Waters J. (2009). Effect of ocean acidification on the speciation of metals in seawater. Oceanography 22, 72–85. 10.5670/oceanog.2009.98

[B52] Monnin L. Ciffroy P. Garnier J. M. Ambrosi J.-P. Radakovitch O. (2018). Remobilization of trace metals during laboratory resuspension of contaminated sediments from a dam reservoir. J. Soils Sediments 18, 2596–2613. 10.1007/s11368-018-1931-5

[B53] Moon H. S. Komlos J. Jaffé P. R. (2007). Uranium reoxidation in previously bioreduced sediment by dissolved oxygen and nitrate. Environ. Sci. Technol. 41, 4587–4592. 10.1021/es063063b 17695901

[B54] Naifar I. Pereira F. Zmemla R. Bouaziz M. Elleuch B. Garcia D. (2018). Spatial distribution and contamination assessment of heavy metals in marine sediments of the southern coast of Sfax, Gabes Gulf, Tunisia. Mar. Pollut. Bull. 131, 53–62. 10.1016/j.marpolbul.2018.03.048 29886979

[B55] O'Sullivan L. A. Rinna J. Humphreys G. Weightman A. J. Fry J. C. (2006). Culturable phylogenetic diversity of the phylum “*Bacteroidetes”* from river epilithon and coastal water and description of novel members of the family *Flavobacteriaceae: Epilithonimonas tenax* gen. nov., sp. nov. and *Persicivirga xylanidelens* gen. nov., sp. Int. J. Syst. Evol. Microbiol. 56, 169–180. 10.1099/ijs.0.63941-0 16403883

[B56] Peng J. F. Song Y. H. Yuan P. Cui X. Y. Qiu G. L. (2009). The remediation of heavy metals contaminated sediment. J. Hazard. Mater. 161, 633–640. 10.1016/j.jhazmat.2008.04.061 18547718

[B57] Quéméneur M. Garrido F. Billard P. Breeze D. Leyval C. Jauzein M. (2016). Bacterial community structure and functional *arrA* gene diversity associated with arsenic reduction and release in an industrially contaminated soil. Geomicrobiol. J. 33, 839–849. 10.1080/01490451.2015.1118167

[B58] Rabaoui L. El Zrelli R. Ben Mansour M. Balti R. Mansour L. Tlig-Zouari S. (2015). On the relationship between the diversity and structure of benthic macroinvertebrate communities and sediment enrichment with heavy metals in Gabes Gulf, Tunisia. J. Mar. Biol. Assoc. 95, 233–245. 10.1017/S0025315414001489

[B59] Reijonen I. Metzler M. Hartikainen H. (2016). Impact of soil pH and organic matter on the chemical bioavailability of vanadium species: the underlying basis for risk assessment. Environ. Pollut. 210, 371–379. 10.1016/j.envpol.2015.12.046 26807983

[B60] Rekik A. Drira Z. Guermazi W. Elloumi J. Maalej S. Aleya L.. (2012). Impacts of an uncontrolled phosphogypsum dumpsite on summer distribution of phytoplankton, copepods and ciliates in relation to abiotic variables along the near-shore of the southwestern Mediterranean coast. Mar. Pollut. Bull. 64, 336–346. 10.1016/j.marpolbul.2011.11.005 22154276

[B61] Ross S. (1994). Toxic Metals in Soil-Plant Systems. Chichester: Wiley.

[B62] Sammari C. Koutitonsky V. G. Moussa M. (2006). Sea level variability and tidal resonance in the Gulf of Gabes, Tunisia. Cont. Shelf Res. 26, 338–350. 10.1016/j.csr.2005.11.006

[B63] Saulnier I. Mucci A. (2000). Trace metal remobilization following the resuspension of estuarine sediments: Saguenay Fjord, Canada. Appl. Geochemistry 15, 191–210. 10.1016/S0883-2927(99)00034-7

[B64] Shannon C. E. Weaver W. (1949). The Mathematical Theory of Communication. Champaign, IL; Urbana, IL: University of Illinois Press.

[B65] Shipley H. J. Gao Y. Kan A. T. Tomson M. B. (2011). Mobilization of trace metals and inorganic compounds during resuspension of Anoxic Sediments from Trepangier Bayou, Louisiana. J. Environ. Qual. 40, 484–491. 10.2134/jeq2009.0124 21520756

[B66] Simpson E. H. (1949). Measurement of diversity. Nature 163:688 10.1038/163688a0

[B67] Sun M. Y. Dafforn K. A. Brown M. V. Johnston E. L. (2012). Bacterial communities are sensitive indicators of contaminant stress. Mar. Pollut. Bull. 64, 1029–1038. 10.1016/j.marpolbul.2012.01.035 22385752

[B68] Tabak H. H. Lens P. Van Hullebusch E. D. Dejonghe W. (2005). Developments in bioremediation of soils and sediments polluted with metals and radionuclides - 1. Microbial processes and mechanisms affecting bioremediation of metal contamination and influencing metal toxicity and transport. Rev. Environ. Sci. Biotechnol. 4, 115–156. 10.1007/s11157-005-2169-4

[B69] Tessier E. Garnier C. Mullot J. U. Lenoble V. Arnaud M. Raynaud M.. (2011). Study of the spatial and historical distribution of sediment inorganic contamination in the Toulon bay (France). Mar. Pollut. Bull. 62, 2075–2086. 10.1016/j.marpolbul.2011.07.022 21864863

[B70] Wang K. Zhang D. Xiong J. Chen X. Zheng J. Hu C.. (2015). Response of bacterioplankton communities to cadmium exposure in coastal water microcosms with high temporal variability. Appl. Environ. Microbiol. 81, 231–240. 10.1128/AEM.02562-14 25326310 PMC4272717

[B71] Wang Z. Wang Y. Zhao P. Chen L. Yan C. Yan Y.. (2015). Metal release from contaminated coastal sediments under changing pH conditions: Implications for metal mobilization in acidified oceans. Mar. Pollut. Bull. 101, 707–715. 10.1016/j.marpolbul.2015.10.026 26481412

[B72] White C. Sayer I. A. Gadd G. M. (1997). Microbial solubilization and immobilization of toxic metals: key biogeochemical processes for treatment of contamination. FEMS Microbiol Rev. 20, 503–516. 10.1111/j.1574-6976.1997.tb00333.x 9299717

[B73] Witt V. Wild C. Anthony K. R. N. Diaz-Pulido G. Uthicke S. (2011). Effects of ocean acidification on microbial community composition of, and oxygen fluxes through, biofilms from the Great Barrier Reef. Environ. Microbiol. 13, 2976–2989. 10.1111/j.1462-2920.2011.02571.x 21906222

[B74] Wu Y. Zeng J. Zhu Q. Zhang Z. Lin X. (2017). pH is the primary determinant of the bacterial community structure in agricultural soils impacted by polycyclic aromatic hydrocarbon pollution. Sci. Rep. 7:40093. 10.1038/srep40093 28051171 PMC5209717

[B75] Xu W. Li X. Wai O. W. H. Huang W. Yan W. (2015). Remobilization of trace metals from contaminated marine sediment in a simulated dynamic environment. Environ. Sci. Pollut. Res. 22, 19905–19911. 10.1007/s11356-015-5228-6 26289335

[B76] Yamazaki I. M. Geraldo L. P. (2003). Uranium content in phosphate fertilizers commercially produced in Brazil. Appl. Radiat. Isot. 59, 133–136. 10.1016/S0969-8043(03)00159-3 12941502

[B77] Zaghden H. Serbaji M. M. Saliot A. Sayadi S. (2016). The Tunisian Mediterranean coastline: potential threats from urban discharges Sfax-Tunisian Mediterranean coasts. Desalin. Water Treat. 57, 24765–24777. 10.1080/19443994.2016.1149107

[B78] Zouch H. Karray F. Armougom F. Chifflet S. Hirschler-Réa A. Kharrat H.. (2017). Microbial diversity in sulfate-reducing marine sediment enrichment cultures associated with anaerobic biotransformation of coastal stockpiled phosphogypsum (Sfax, Tunisia). Front. Microbiol. 8:1583. 10.3389/fmicb.2017.01583 28871244 PMC5566975

