# Supplementary material for: Effect of Acidic Industrial Effluent Release on Microbial Diversity and Trace Metal Dynamics During Resuspension of Coastal Sediment
Source: Front Microbiol. 2018 Dec 14;9:3103. doi: 10.3389/fmicb.2018.03103 (PMC6302000; doi:10.3389/fmicb.2018.03103)
Supplement: Supplementary file 1 [file Table_1.docx]

**Table S1. Chemical properties of seawater (SW) and acid wastewater (AWW) collected on the Sfax coast (Tunisia, South Mediterranean Sea).** Values are means of data from three samples ± confidence intervals (error bars).

|  | SW | AWW |
| --- | --- | --- |
| pH *in situ* | 8.3 ± 0.2 | 2.4 ± 0.1 |
| Temperature *in situ* (°C) | 31.5 ± 0.5 | 24.5 ± 0.0 |
| Eh *in situ* (mV) | 280 | 289 |
| PO_4_^3-^ (µM) | 6.4−82.1^1^ | 70842^2^ |
| Trace element concentrations (µg.L^-1^) | | |
| As | 3.37 ± 0.20 | 20.60 ± 0.90 |
| Cd | 0.15 ± 0.01 | 181.50 ± 10.60 |
| Co | 0.72 ± 0.10 | 7.40 ± 0.40 |
| Cr | 0.41 ± 0.06 | 1234.70 ± 70.50 |
| Mn | 21.85 ± 1.63 | 228.00 ± 13.70 |
| Mo | 19.45 ± 0.03 | 6.40 ± 0.30 |
| Ni | 11.68 ± 1.64 | 113.00 ± 5.20 |
| Pb | 0.07 ± 0.02 | 1.41 ± 0.20 |
| Sb | 0.82 ± 0.01 | 3.80 ± 0.30 |
| Ti | 0.70 ± 0.25 | 355.10 ± 25.40 |
| U | 4.79 ± 0.04 | 3.10 ± 0.20 |
| V | 5.92 ± 1.04 | 321.90 ± 21.50 |

^1^ Data obtained from Fourati et al. (2018)

^2^ Data obtained from Melki and Gueddari (2018)

**Table S2. Multi-collinearity test between abiotic variables using cross-correlations among 8 variables obtained during sediment resuspension experiments.** The highest correlations (≥ 0.9) are indicated in bold.

| Pearson coefficient (r) | | | | | | | | |
| --- | --- | --- | --- | --- | --- | --- | --- | --- |
|  | Time | OD | pH | Cd | U | As | V | AWW |
| Time | 1.00 |  |  |  |  |  |  |  |
| OD | 0.48 | 1.00 |  |  |  |  |  |  |
| pH | 0.19 | 0.58 | 1.00 |  |  |  |  |  |
| Cd | **0.94** | 0.35 | 0.02 | 1.00 |  |  |  |  |
| U | 0.17 | 0.43 | 0.85 | -0.06 | 1.00 |  |  |  |
| As | -0.55 | -0.41 | -0.04 | -0.58 | 0.23 | 1.00 |  |  |
| V | 0.42 | 0.01 | -0.62 | 0.53 | -0.57 | -0.30 | 1.00 |  |
| AWW | 0.00 | -0.38 | **-0.90** | 0.22 | **-0.97** | -0.26 | 0.72 | 1.00 |
| P-value | | | | | | | | |
| Time |  |  |  |  |  |  |  |  |
| OD | 0.0186 |  |  |  |  |  |  |  |
| pH | 0.3755 | 0.0030 |  |  |  |  |  |  |
| Cd | 0.0000 | 0.0975 | 0.9442 |  |  |  |  |  |
| U | 0.4232 | 0.0369 | 0.0000 | 0.7868 |  |  |  |  |
| As | 0.0051 | 0.0494 | 0.8605 | 0.0031 | 0.2814 |  |  |  |
| V | 0.0421 | 0.9694 | 0.0012 | 0.0074 | 0.0040 | 0.1594 |  |  |
| AWW | 1.0000 | 0.0674 | 0.0000 | 0.2925 | 0.0000 | 0.2147 | 0.0000 |  |
|  |  |  |  |  |  |  |  |  |

**Table S3. Richness, diversity and abundance of microbial communities in coastal sediment (SED), seawater (SW), acid wastewater (AWW), and sediment samples collected at 4 different times (T3, T5, T7 and T10 days) from biotic incubations in P or S conditions (with or without AWW addition, respectively).** Values are means of data from two initial samples or three incubation samples ± confidence intervals (error bars).

| Sample | Sequence Number^1^ | OTU Number | Abundant OTU Number^3^ | Chao1 index | Coverage (%) | Simpson index | Shannon index | PD whole tree | Bacterial abundance (16S rDNA copies.g^-1^ or L^-1^) |
| --- | --- | --- | --- | --- | --- | --- | --- | --- | --- |
| SW | 26735.5 ±784.5 | 1068 ± 26 | 15.0 ± 0.0 | 1234.7 ± 17.6 | 86.48 ± 0.87 | 0.99 ± 0.05 | 7.68 ± 0.18 | 63.83 ± 1.23 | 7.75 ± 0.77 x 10^8^ |
| SED | 33236.5 ±720.5 | 1041 ± 6 | 9.5 ± 0.5 | 1227.4 ± 39.1 | 84.88 ± 2.21 | 0.96 ± 0.00 | 7.35 ± 0.04 | 65.12 ± 0.22 | 2.87 ± 0.96 x 10^9^ |
| AWW^2^ | 30997 | 651 | 13 | 990.7 | 65.71 | 0.97 ± 0.00 | 6.79 | 46.6 | 4.97 x 10^6^ |
| S T3 | 24405.0 ± 5343.0 | 1083 ± 5 | 12.3 ± 1.5 | 1224.2 ± 9.8 | 88.47 ± 0.41 | 0.98 ± 0.00 | 7.56 ± 0.08 | 63.40 ± 0.94 | 2.21 ± 0.18 x 10^8^ |
| S T5 | 35503 ± 4077 | 1102 ± 14 | 12.3 ± 1.5 | 1240.6 ± 15.4 | 88.82 ± 1.50 | 0.98 ± 0.01 | 7.64 ± 0.21 | 64.96 ± 0.99 | 2.68 ± 0.25 x 10^8^ |
| S T7 | 25093 ± 3061 | 1108 ± 32 | 12.3 ± 1.5 | 1259.1 ± 23.1 | 88.00 ± 0.90 | 0.98 ± 0.00 | 7.79 ± 0.12 | 65.31 ± 1.94 | 2.61 ± 0.18 x 10^8^ |
| S T10 | 25313 ± 4053 | 1113 ± 10 | 13.7 ± 2.3 | 1271.1 ± 4.6 | 87.54 ± 1.01 | 0.98 ± 0.01 | 7.76 ± 0.05 | 65.56 ± 0.76 | 1.77 ± 0.14 x 10^8^ |
| P T3 | 36792 ± 2471 | 1072 ± 12 | 13.3 ± 1.5 | 1258.1 ± 32.8 | 85.26 ± 1.77 | 0.97 ± 0.00 | 7.34 ± 0.15 | 62.29 ± 0.32 | 5.10 ± 0.15 x 10^8^ |
| PT5 | 28347 ± 3100 | 1042 ± 27 | 12.3 ± 2.5 | 1198.8 ± 26.3 | 86.94 ± 1.95 | 0.97 ± 0.00 | 7.34 ± 0.21 | 62.56 ± 2.28 | 1.10 ± 0.11 x 10^8^ |
| P T7 | 23122 ± 3994 | 1079 ± 23 | 13.3 ± 3.3 | 1229.4 ± 16.6 | 87.80 ± 1.74 | 0.97 ± 0.01 | 7.46 ± 0.38 | 64.10 ± 1.66 | 1.82 ± 0.07 x 10^8^ |
| P T10 | 31638 ± 3730 | 1065 ± 19 | 13.3 ± 2.3 | 1217.4 ± 17.6 | 87.53 ± 0.78 | 0.97 ± 0.01 | 7.48 ± 0.19 | 63.41 ± 1.58 | 2.50 ± 0.27 x 10^8^ |

^1^ Number of sequences analyzed by QIIME after pretreatment of raw sequences

^2^ Data obtained for single DNA sample from one AWW filter due to PCR failure with second AWW DNA

^3^ Abundant OTU corresponds to an OTU that reached > 1% of all sequences

**Table S4. Blast analysis on the dominant OTU (>1% of total sequences) obtained from microbial communities in the sediment (SED) of Sfax coast (Tunisia, South Mediterranean Sea).**

| OTU no. [GenBank number] | OTU frequency (%)^1^ | Closest cultivated relative retrieved from NCBI nucleotide database | | |
| --- | --- | --- | --- | --- |
|  |  | Taxonomy (phylum/classe ; order) | Species (accession number) | Identity (%) |
| 593437 [22696] | 3.90 ± 0.22 | *Alphaproteobacteria; Rhodobacterales* | *Thioclava atlantica* (NR_136453) | 98 |
| 4390055 [MH002289] | 16.33 ± 0.60 | *Alphaproteobacteria; Rhodobacterales* | *Thioclava indica* (NR_136454) | 99 |
| 206 [MH002299] | 1.82 ± 0.20 | *Betaproteobacteria; Rhodocyclales* | *Azoarcus toluclasticus* (NR_024970) | 95 |
| 153118 [MH002297] | 1.54 ± 0.12 | *Epsilonproteobacteria* | *Sulfurovum lithotrophicum* (NR_024802) | 98 |
| 584500 [MH002298] | 2.12 ± 0.14 | *Epsilonproteobacteria; Campylobacterales* | *Arcobacter cryaerophilus* (NR_025905) | 99 |
| 265 [MH002301] | 1.66 ± 0.13 | *Gammaproteobacteria; Methylococcales* | *Methylocaldum szegediense* (NR_026064) | 88 |
| 241 [MH002300] | 1.32 ± 0.30 | *Bacteroidia; Bacteroidales;* | *Uncultured Bacteroidales bacterium* (KC006386) | 95 |
| 266 [MH002271] | 7.36 ± 0.74 | *Flavobacteriia; Flavobacteriales* | *Gaetbulibacter jejuensis* (NR_116705) | 96 |
| 66 [MH002283] | 1.32 ± 0.01 | *Flavobacteriia; Flavobacteriales* | *Namhaeicola litoreus* (NR_132282) | 95 |

^1^Values are means of sequence frequency in two sediment samples ± confidence intervals (error bars).

**Table S5. Blast analysis on the dominant OTU (>1% of total sequences) obtained from microbial communities in the seawater (SW) of Sfax coast (Tunisia, South Mediterranean Sea).**

| OTU no. [GenBank number] | OTU frequency (%)^1^ | Closest cultivated relative retrieved from NCBI nucleotide database | | |
| --- | --- | --- | --- | --- |
|  |  | Taxonomy (phylum/classe ; order) | Species (accession number) | Identity (%) |
| 539299 [MH002254] | 1.13 ± 0.01 | *Alphaproteobacteria; Rhodobacterales* | *Marivita hallyeonensis* (NR_109362) | 99 |
| 435644 [MH002285] | 1.17 ± 0.12 | *Alphaproteobacteria; Rhodobacterales* | *Aestuariivita boseongensis* (NR_133957) | 99 |
| 562126 [MH002286] | 1.07 ± 0.17 | *Alphaproteobacteria; Rhodobacterales* | *Cribrihabitans marinus* (NR_133733) | 98 |
| 346431 [MH002287] | 8.75 ± 1.59 | *Alphaproteobacteria; Rhodobacterales* | *Cribrihabitans marinus* (NR_133733) | 98 |
| 829814 [MH002288] | 1.43 ± 0.04 | *Alphaproteobacteria; Rhodobacterales* | *Tropicibacter multivorans* (NR_108509) | 98 |
| 4390055 [MH002289] | 1.27 ± 0.11 | *Alphaproteobacteria; Rhodobacterales* | *Thioclava indica* (NR_136454) | 99 |
| 369920 [MH002290] | 6.00 ± 1.01 | *Alphaproteobacteria; Rhodobacterales* | *Cribrihabitans marinus* (NR_133733) | 98 |
| 1107606 [MH002291] | 1.49 ± 0.1 | *Alphaproteobacteria; Rhodobacterales* | *Mameliella atlantica* (NR_136489) | 98 |
| 1016465 [MH002252] | 1.94 ± 0.31 | *Alphaproteobacteria; Pelagibacterales* | *Candidatus Pelagibacter ubique* (SAR11) (NR_074224) | 99 |
| 404788 [MH002292] | 3.99 ± 0.45 | *Cyanobacteria; Synechococcales* | *Synechococcus rubescens* (NR_125481) | 97 |
| 557211 [MH002293] | 1.94 ± 0.12 | *Cyanobacteria; Synechococcales* | *Prochlorococcus marinus* (NR_125480) | 97 |
| 572032 [MH002294] | 2.89 ± 0.23 | *Actinobacteria; Micrococcales* | *Candidatus Limnoluna rubra* (NR_125497) | 99 |
| 232 [MH002295] | 2.57 ± 0.09 | *Actinobacteria; Micrococcales* | *Pontimonas salivibrio* (NR_109611) | 97 |
| 106 [MH002273] | 2.32 ± 0.19 | *Flavobacteriia; Flavobacteriales* | *Winogradskyella pulchriflava* (NR_109526) | 95 |

^1^Values are means of sequence frequency in two seawater samples ± confidence intervals (error bars).

**Table S6. Blast analysis on the dominant OTU (>1% of total sequences) obtained from acid wastewater (AWW) of Sfax coast (Tunisia).**

| OTU no. [GenBank number] | OTU frequency (%) | Closest cultivated relative retrieved from NCBI nucleotide database | | |
| --- | --- | --- | --- | --- |
|  |  | Taxonomy (phylum/classe ; order) | Species (accession number) | Identity (%) |
| 968336 [MH002302] | 9.58 | *Bacilli; Bacillales* | *Tumebacillus ginsengisoli* (NR_112564*)* | 96 |
| 4298525 [MH002303] | 8.78 | *Bacilli; Bacillales* | *Tumebacillus ginsengisoli* (NR_112564) | 95 |
| 780650 [MH002304] | 2.35 | *Clostridia; Clostridiales* | *Clostridium saudii* (NR_144696) | 98 |
| 606927 [MH002305] | 1.11 | *Clostridia; Clostridiales* | *Intestinibacter bartlettii* (NR_027573) | 99 |
| 555945 [MH002306] | 6.09 | *Clostridia; Clostridiales* | *Romboutsia timonensis* (NR_144740) | 99 |
| 338222 [MH002307] | 1.00 | *Clostridia; Clostridiales* | *Clostridium saudii* (NR_144696) | 99 |
| 346431 [MH002287] | 1.20 | *Alphaproteobacteria; Rhodobacterales* | *Cribrihabitans marinus* (NR_133733) | 98 |
| 369920 [MH002290] | 1.28 | *Alphaproteobacteria; Rhodobacterales* | *Cribrihabitans marinus* (NR_133733*)* | 98 |
| 532994[MH002308] | 1.15 | *Gammaproteobacteria; Alteromonadales* | *Pseudoalteromonas haloplanktis* (NR_044837) | 99 |
| 1074625 [MH002309] | 6.11 | *Actinobacteria; Micrococcales* | *Candidatus Rhodoluna limnophila* (NR_125490) | 98 |
| 232 [MH002295] | 1.37 | *Actinobacteria; Micrococcales* | *Chryseoglobus frigidaquae* (NR_115999) | 97 |
| 50167 [MH002210] | 1.17 | *Deltaaproteobacteria ; Desulfobacterales* | *Desulforhopalus singaporensis* (NR_028742) | 96 |
| 404788 [MH002292] | 1.32 | *Cyanobacteria; Synechococcales* | *Synechococcus rubescens* (NR_125481) | 97 |

**Table S7. Results of the BioEnv analysis for the relationship between the microbial community and the abiotic variables obtained from the sediment resuspension experiments.** Spearman correlation for the best model is shown in bold.

| Model | Parameters | Correlation | p-value |
| --- | --- | --- | --- |
| 1 | pH | 0.4997 | 0.002 |
| **2** | **pH, Cd** | **0.6265** | **0.002** |
| 3 | pH, Cd, U | 0.6085 | 0.002 |
| 4 | pH, Cd, U, As | 0.5625 | 0.002 |
| 5 | pH, Cd, U, As, V | 0.4852 | 0.002 |

**Table S8. Blast analysis on the 32 representative OTU obtained from the biotic incubations of sediment resuspensions and selected by Principal Coordinate Analysis (PCoA).**

| OTU no. [GenBank number] | Closest cultivated relative retrieved from NCBI nucleotide database | | | Source of isolation |
| --- | --- | --- | --- | --- |
|  | Taxonomy (phylum/classe ; order) | Species (accession number) | Identity (%) |  |
| 1016465 [MH002252] | *Alphaproteobacteria; Pelagibacterales ; Pelagibacteraceae* | *Candidatus Pelagibacter sp.* (LN850161*)* | 99 | Red sea water surface |
| 539299 [MH002254] | *Alphaproteobacteria; Rhodobacterales;Rhodobacteraceae* | *Marivita hallyeonensis* (NR_109362) | 99 | Seawater |
| 251 [MH002267] | *Alphaproteobacteria;Rhodobacterales;Rhodobacteraceae* | *Shimia sagamensis* (NR_137204) | 87 | Cold-seep sediment |
| 536800 [MH002261] | *Bacteroidetes Order II. Incertae sedis; Rhodothermaceae* | *Unclassified Rhodothermaceae* (KU517707) | 90 | Saline hot spring |
| 268113 [MH002282] | *Bacteroidetes Order II. Incertae sedis; Rhodothermaceae* | *Unclassified Rhodothermaceae* (KU517707) | 89 | Saline hot spring |
| 274874 [MH002258] | *Balneolia; Balneolales; Balneolaceae* | *Gracilimonas tropica* (NR_044361) | 92 | Cyanobacterial culture from tropical Pacific Ocean surface water |
| 210 [MH002284] | *Caldilineae; Caldilineales; Caldilineaceae* | *Litorilinea aerophila* (NR_132330) | 90 | Intertidal hot spring |
| 356532 [MH002275] | *Cytophagia* | *Unclassified Cytophagia* (JX439384) | 95 | Sea water |
| 73722 [MH002255] | *Gammaproteobacteria; Alteromonadales; Alteromonadaceae* | *Alteromonas hispanica* (KF424806) | 99 | Deep-sea sediments |
| 823476 [MH002257] | *Gammaproteobacteria; Alteromonadales; Alteromonadaceae* | *Alteromonas macleodii* (KY382792 ) | 99 | Marine hydroid polyp Hydractinia echinata |
| 140 [MH002272] | *Gammaproteobacteria; Oceanospirillales* | *Bermanella marisrubri* (NR_042750*)* | 90 | Red Sea |
| 7 [MH002266] | *Gammaproteobacteria* | *Pseudohongiella spirulinae* (NR_126265) | 93 | Cultivation pond of Spirulina platensis in Sanya |
| 105 [MH002274] | *Gammaproteobacteria; Oceanospirillales; Alcanivoracaceae* | *Marinicella litoralis* (NR_112913) | 97 | Coastal seawater |
| 304039 [MH002277] | *Gammaproteobacteria; Chromatiales; Wenzhouxiangellaceae* | *Wenzhouxiangella marina* (CP012154) | 98 | Indian Ocean |
| 66 [MH002283] | *Flavobacteriia; Flavobacteriales; Flavobacteriaceae* | *Namhaeicola litoreus* (NR_132282) | 95 | Seawater |
| 1657387  [MH002311] | *Flavobacteriia; Flavobacteriales; Flavobacteriaceae* | *Flavobacterium haoranii* (NR_117422) | 95 | Wastewater treatment system |
| 168126 [MH002262] | *Flavobacteriia; Flavobacteriales; Flavobacteriaceae* | *Olleya marilimosa* (FJ161232) | 97 | Shandong coast, China |
| 109 [MH002269] | *Flavobacteriia; Flavobacteriales; Flavobacteriaceae* | *Namhaeicola litoreus* (NR_132282) | 94 | Seawater |
| 165 [MH002270] | *Flavobacteriia; Flavobacteriales; Flavobacteriaceae* | *Tenacibaculum skagerrakense* (KU051415) | 95 | Seawater |
| 266 [MH002271] | *Flavobacteriia; Flavobacteriales; Flavobacteriaceae* | *Gaetbulibacter jejuensis* (NR_116705) | 96 | Seawater |
| 1 [MH002264] | *Flavobacteriia; Flavobacteriales; Flavobacteriaceae* | *Tenacibaculum xiamenense* (NR_109729) | 97 | Coastal seawater |
| 106 [MH002273] | *Flavobacteriia; Flavobacteriales; Flavobacteriaceae* | *Winogradskyella pulchriflava* (NR_109526) / *Sediminibacter furfurosus* (NR_041452) | 95 | Marine sediment |
| 278602 [MH002276] | *Flavobacteriia; Flavobacteriales; Flavobacteriaceae* | *Winogradskyella litoriviva* (NR_137338) | 98 | Coastal seawater |
| 125 [MH002278] | *Flavobacteriia; Flavobacteriales; Flavobacteriaceae* | *Winogradskyella litoriviva* (NR_137338) | 98 | Coastal seawater |
| 205 [MH002263] | *Saprospiria; Saprospirales; Haliscomenobacteraceae* | *Phaeodactylibacter xiamenensis* (NR_134132) | 89 | Marine alga Phaeodactylum tricornutum |
| 273031 [MH002259] | *Saprospiria; Saprospirales; Lewinellaceae* | *Lewinella nigricans* (NR_115013) | 99 | Beach sediment |
| 3 [MH002265] | *Saprospiria; Saprospirales; Lewinellaceae* | *Lewinella cohaerens*( KF228160) | 92 | Aerated compost tea from crop residues compost |
| 30 [MH002268] | *Saprospiria; Saprospirales; Haliscomenobacteraceae* | *Portibacter lacus* (NR_113569) | 91 | Saline lake |
| 114 [MH002279] | *Saprospiria; Saprospirales; Lewinellaceae* | *Lewinella cohaerens* (KF228160) | 98 | Aerated compost tea from crop residues compost |
| 3074191 [MH002260] | *Verrucomicrobia* | *Verrucomicrobia bacterium* (JF488486) | 96 | Gulf of Maine |
| 787618 [MH002256] | *Verrucomicrobia; unclassified Verrucomicrobia* | *Unclassified Verrucomicrobia* (KP030837*)* | 96 | Coastal seawater |
| 3180 [MH002281] | *Verrucomicrobia* | *Unclassified Verrucomicrobia* (KP030837) | 95 | Coastal seawater |

**Table S9. Spearman’s rank correlation coefficients between dissolved concentrations of four trace elements (As, Cd, U, V) and relative abundance of representative OTU (selected from the PCoA) of sediment resuspension experiments.** Values in bold are significant at p$\leq$ 0.05. Correlation with *Bacteroidetes* OTUs are underlined in grew.

| TE  OTU | Cd | U | As | V |
| --- | --- | --- | --- | --- |
| 1^1^ | 0.6190 | -0.3333 | -0.2619 | **0.8095** |
| 168126^1^ | **0.8333** | -0.1190 | -0.5476 | **0.8333** |
| 66^1^ | **0.7857** | 0.0714 | -0.6429 | 0.6429 |
| 109^1^ | **0.8095** | -0.2381 | -0.5000 | **0.9048** |
| 165^1^ | **0.9102** | 0.1317 | -0.6108 | 0.7066 |
| 106^1^ | 0.2381 | **-0.7381** | -0.2143 | **0.7857** |
| 266^1^ | 0.7143 | -0.2857 | -0.4762 | **0.8571** |
| 125^1^ | **-0.8333** | 0.1190 | 0.6190 | **-0.8333** |
| 278602 ^1^ | **-0.8333** | 0.1190 | 0.4286 | **-0.8333** |
| 1657387^1^ | -0.4286 | **-0.8571** | 0.5000 | 0.2857 |
| 268113^2^ | **0.9524** | 0.2619 | -0.5476 | 0.6429 |
| 536800^2^ | **0.8333** | **0.7381** | -0.5952 | 0.1667 |
| 3^3^ | **0.8333** | **0.7381** | -0.5952 | 0.1667 |
| 30^3^ | 0.5714 | **0.9286** | -0.5000 | -0.2143 |
| 205^3^ | 0.5714 | **0.9286** | -0.5000 | -0.2143 |
| 273031^3^ | **0.8810** | 0.5476 | -0.6429 | 0.3571 |
| 114^3^ | 0.5238 | **0.9048** | -0.4762 | -0.2381 |
| 356532^4^ | -0.2381 | **0.7381** | 0.0000 | **-0.7857** |
| 1016465^5^ | 0.1905 | **0.9524** | -0.2619 | -0.5714 |
| 539299^5^ | **-0.8571** | 0.0000 | 0.6905 | **-0.7619** |
| 251^5^ | **-0.8333** | **-0.7381** | 0.5952 | -0.1667 |
| 7^6^ | **0.9286** | 0.5714 | -0.6667 | 0.3810 |
| 140^6^ | **0.7619** | -0.2619 | -0.5000 | **0.8810** |
| 105^6^ | 0.0476 | **0.8810** | -0.3333 | -0.6429 |
| 304039^6^ | 0.0000 | **0.8571** | -0.3095 | -0.6667 |
| 823476^6^ | -0.5714 | 0.1429 | 0.2381 | -0.6190 |
| 73722^6^ | -0.5238 | -0.0476 | 0.1190 | -0.4286 |
| 210^7^ | **0.9701** | 0.2156 | -0.5629 | 0.6946 |
| 274874^7^ | 0.2381 | **0.9762** | -0.2857 | -0.5476 |
| 787618^8^ | 0.5952 | **0.8333** | -0.4524 | -0.1190 |
| 3074191^8^ | **-0.7381** | -0.6905 | 0.6905 | -0.1190 |
| 3180^8^ | 0.3095 | **0.9048** | -0.4524 | -0.4286 |

^1^ OTU belonged to *Flavobacteriaceae* family (p_*Bacteroidetes*)

^2^ OTU belonged to *Rhodothermaceae* family (p_*Bacteroidetes*)

^3^ OTU belonged to *Saprospirales* order (p_*Bacteroidetes*)

^4^ OTU belonged to *Cytophagia* class (p_*Bacteroidetes*)

^5^ OTU belonged to *Alphaproteobacteria* class

^6^ OTU belonged to *Gammaproteobacteria* class

^7^ OTU belonged to *Balneolaceae* family

^8^ OTU belonged to *Verrucomicrobia* class
